# Supplementary material for: Quantum Artificial Neural Network Approach to Derive a Highly Predictive 3D-QSAR Model for Blood–Brain Barrier Passage
Source: Int J Mol Sci. 2021 Oct 12;22(20):10995. doi: 10.3390/ijms222010995 (PMC8537149; doi:10.3390/ijms222010995)
Supplement: Supplementary file 1 [file ijms-22-10995-s001.zip › ijms-1355673-supplementary.pdf]

International Journal of
Molecular Sciences

MDPI

Supplementary Material

Quantum Artificial Neural Network Approach to Derive a Highly Predictive 3D-QSAR Model for Blood–Brain Barrier Passage

Taeho Kim,[1] Byoung Hoon You,[2] Songhee Han,[2] Ho Chul Shin,[2] Kee-Choo Chung,[1,*] Hwangseo Park[1,*]

[1] Department of Bioscience and Biotechnology, Sejong University, 209 Neungdong-ro, Kwangjin-gu, Seoul 05006, Korea
[2] Whan In Pharmaceutical Co., Ltd., 11, Beobwon-ro 6-gil, Songpa-gu, Seoul 05855, Korea
* Correspondence: Tel: +82-2-10-2963-1635 (KCC); +82-2-3408-3766 (HP); Fax: +82-2-3408-4334 (KCC); +82-2-3408-4334 (HP); E-mail: kitto28@paran.com (KCC); hspark@sejong.ac.kr (HP)

Supplementary Material

Chemical structures, molecular weights, LogBB values, and PubChem CID of all the molecules in the dataset.	S2-S60, S61-S84
Linear diagrams showing correlation between the experimental and calculated LogBB values of Subset 1-8 molecules in the 3-fold external cross-validation.	

Table S1. Chemical structures, molecular weights, experimental LogBB values, and PubChem CID of all the molecules in the dataset.

Structure	Molecular weight	LogBB	PubChem CID
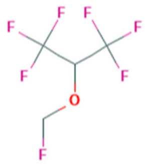	200.053	0.3	5206
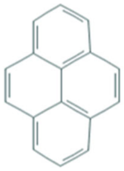	202.255	0.23	31423
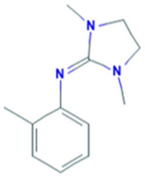	203.2864	-1.39	12032949
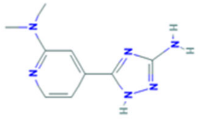	204.234	-1.17	12889418
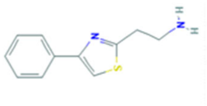	204.2892	-1.3	1497794
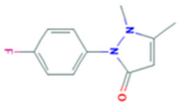	206.2187	-0.05	94957

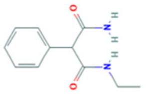	206.2438	-0.19	118753220
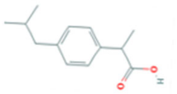	206.284	-0.18	3672
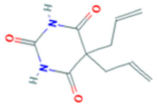	208.2164	-0.22	5842
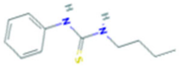	208.3208	0.03	3034183
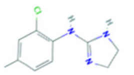	209.6749	-0.65	72138
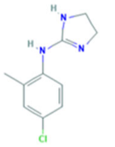	209.6749	-0.87	77870
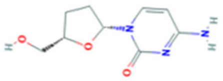	211.22	-1.18	24066

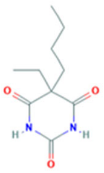	212.248	0.19	6473
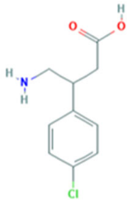	213.6603	-1.52	2284
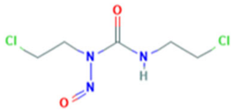	214.045	-0.52	2578
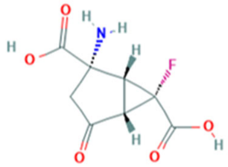	217.1529	-0.41	9837317
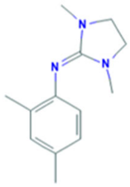	217.3132	-1.3	21675832
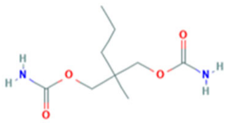	218.2522	-0.15	4064

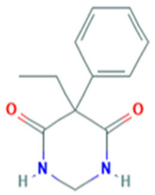	218.2548	-0.07	4909
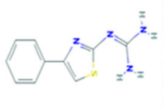	218.2758	-0.18	3763607
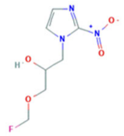	219.1717	-0.01	23274095
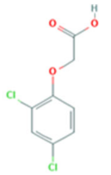	221.0336	0.15	1486
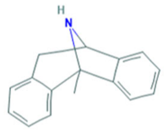	221.3012	1.11	1207
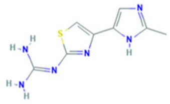	222.2672	-0.04	56051
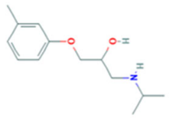	223.3144	0.34	18047

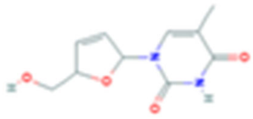	224.2158	-0.48	5155
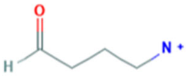	225.2066	-0.84	2022
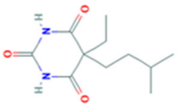	226.2748	0.11	2164
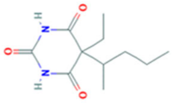	226.2748	0.08	4737
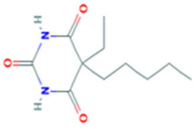	226.2748	0.09	66994
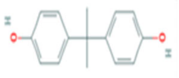	228.2902	-0.12	6623
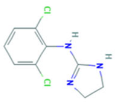	230.0902	0.12	2803

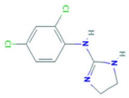	230.0902	0.16	90274
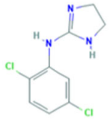	230.0902	0.38	11777724
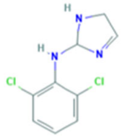	230.0902	0.11	13071367
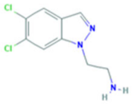	230.0902	0.11	44214615
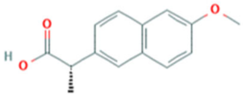	230.2628	0.1	156391
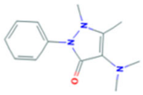	231.2968	0	6009
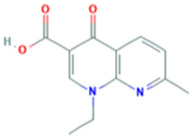	232.2384	-0.66	4421
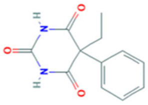	232.2384	0.12	4763

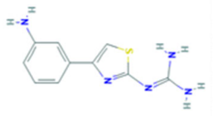	233.2904	-1.15	14022509
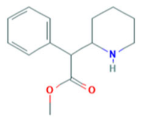	233.3096	0.88	4158
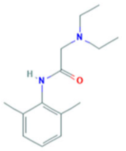	234.3406	0.34	3676
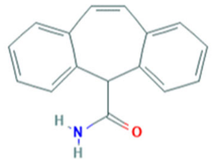	235.2848	0	25255
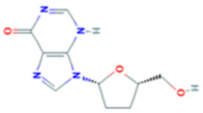	236.2298	-1.301	50599
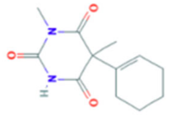	236.27	0.03	3608
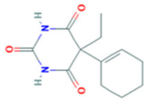	236.27	-0.301	5838

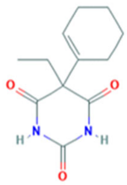	236.2726	-0.04	2554
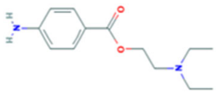	236.3132	0.05	4914
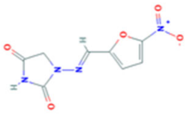	238.1592	-2.09	6604200
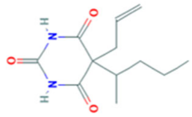	238.2858	0.2	5193
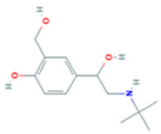	239.3138	-1.14	2083
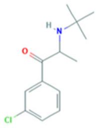	239.7413	1.4	444
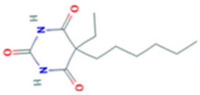	240.3016	0.36	8942

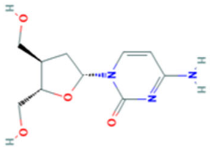	241.2462	-0.79	451819
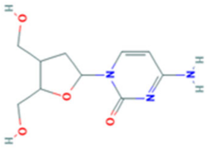	241.2462	-0.79	14840722
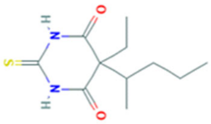	242.3354	-0.19	3000715
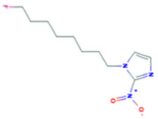	243.2801	-0.17	10377120
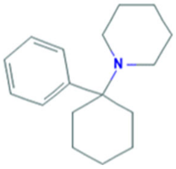	243.3912	0.58	6468
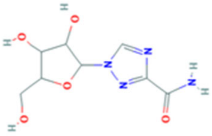	244.2066	-0.67	5064
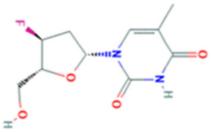	244.2217	-0.59	33039

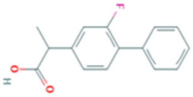	244.2645	-1.68	3394
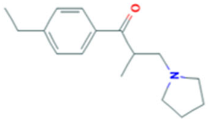	245.3638	1.08	65860
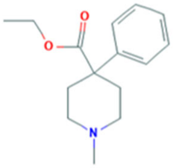	247.3364	0.85	4058
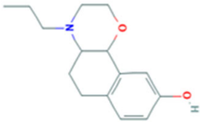	247.3364	0.26	10060538
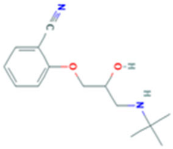	248.3242	0.38	2473
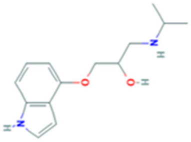	248.3242	-0.14	4828
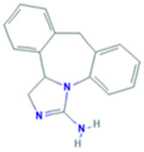	249.3146	-0.16	3241
	249.3522	-0.23	2119

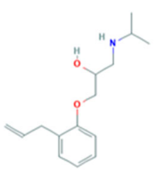			
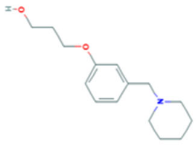	249.3522	-0.02	10444765
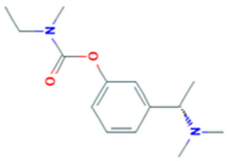	250.34	0.88	77991
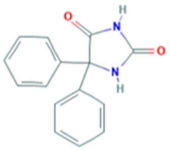	252.272	-0.05	1775
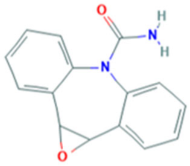	252.272	-0.34	2555
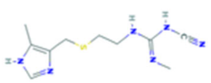	252.3366	-1.06	2756

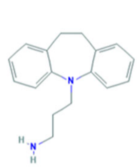	252.3584	1.05	159642
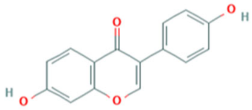	254.2416	-0.15	5281708
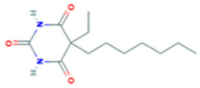	254.3284	0.02	43473
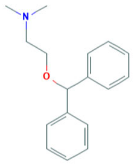	255.359	1.26	3100
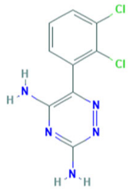	256.0878	0.29	3878
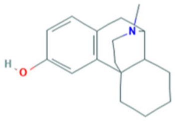	257.3748	0	3918
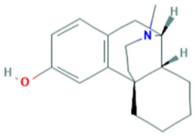	257.3748	0	5359272
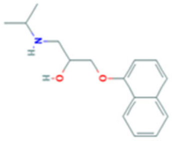	259.3474	0.84	4946

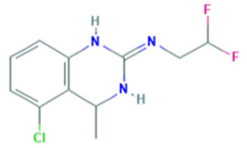	259.6819	0.6	135458393
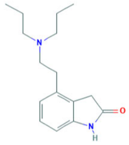	260.3784	0.08	5095
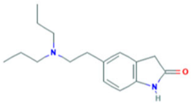	260.3784	0.25	44383796
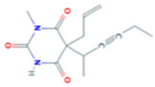	262.3078	-0.07	9034
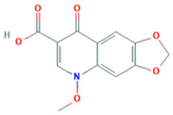	263.2062	-0.92	37614
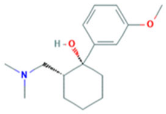	263.379	0.72	33741
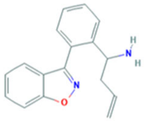	264.3262	0	9861160
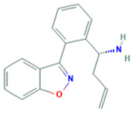	264.3262	0	15524033

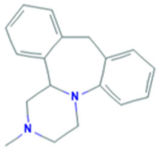	264.3694	0.99	4184
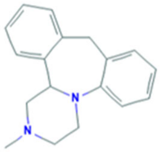	264.3694	0.99	4184
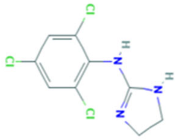	264.5323	0.47	10470115
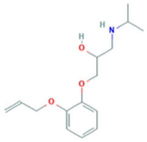	265.3516	0.02	4631
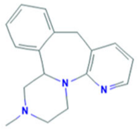	265.3572	0.53	4205
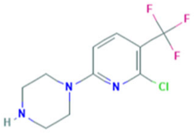	265.661	1.64	9796408
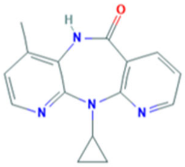	266.3018	0	4463

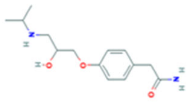	266.3394	-1.14	2249
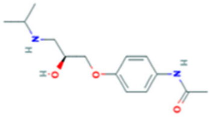	266.3394	-0.55	443371
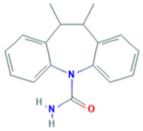	266.342	-0.34	70517986
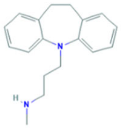	266.3852	1.08	2995
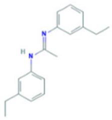	266.3852	1.2	19427054
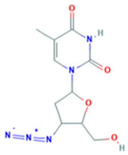	267.2438	-0.74	5726
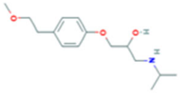	267.3674	1.15	4171

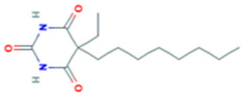	268.3552	0.24	13755681
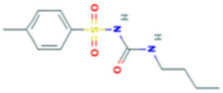	270.3458	-1.01	5505
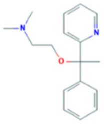	270.3736	0.64	3162
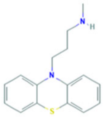	270.3916	0.59	28315
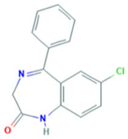	270.7147	0.52	2997
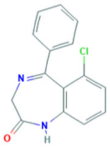	270.7147	0.5	14590445
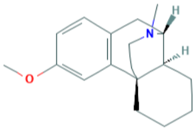	271.4016	0.22	6916184
	271.7427	0.39	14577084

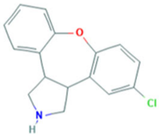			
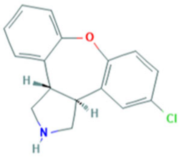	271.7427	0.39	44568616
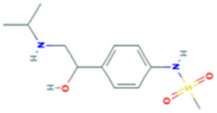	272.3616	-0.28	5253
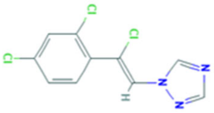	274.5275	0.28	3034012
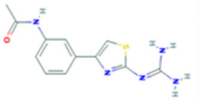	275.3276	-1.57	14022511
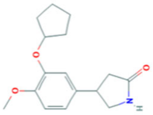	275.3468	0.61	5092
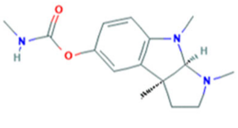	275.3498	0.08	5983
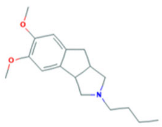	275.39	0.31	6086

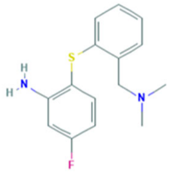	276.3707	0.2	10265481
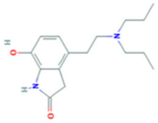	276.3778	-0.43	133741
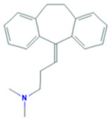	277.4084	0.9	2160
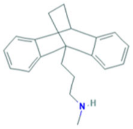	277.4084	1.3	4011
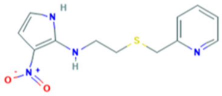	278.3282	-0.66	14022481
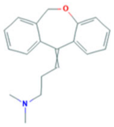	279.381	1	3158
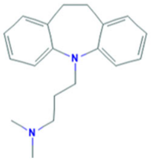	280.412	0.98	3696
	281.3536	0.52	22154175

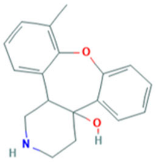			
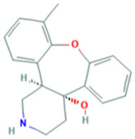	281.3536	0.52	44568615
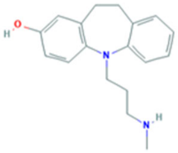	282.3846	0.53	121249
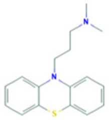	284.4184	1.08	4926
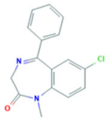	284.7415	0.47	3016
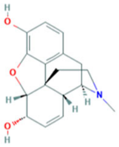	285.342	-0.26	5288826
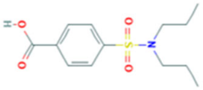	285.3574	-0.4	4911

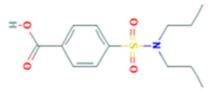	285.3574	-0.4	4911
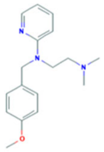	285.3882	0.49	4992
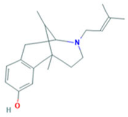	285.4284	0.51	4736
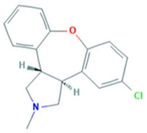	285.7695	1.03	163091
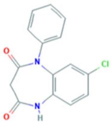	286.7141	0.35	89657
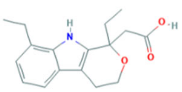	287.3578	-1.42	3308
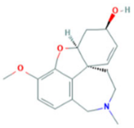	287.3578	0.32	9651

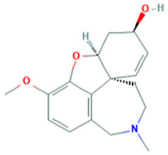	287.3578	0.32	9651
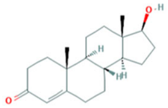	288.429	0	6013
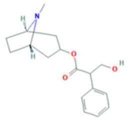	289.3736	-0.06	174174
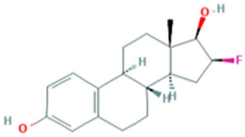	290.3765	-0.3	67101
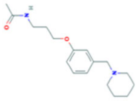	290.4046	-0.46	9971484
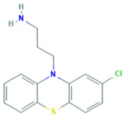	290.8069	0.97	74981
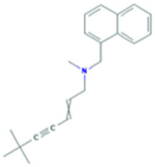	291.4352	0.08	5402

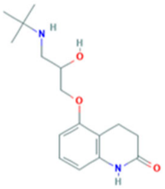	292.3772	0.01	2583
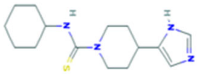	292.4414	-0.17	3035905
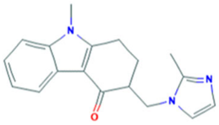	293.3676	0.28	4595
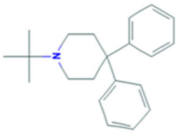	293.451	0.66	68778
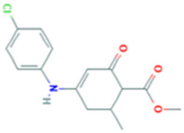	293.7463	-0.95	197322
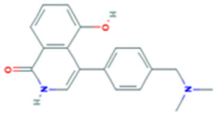	294.3524	-0.09	9839073
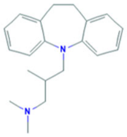	294.4388	0.99	5584

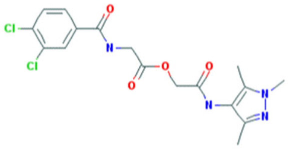	295.3372	-0.34	29939457
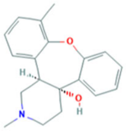	295.3804	0.82	166560
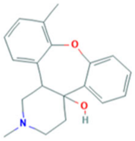	295.3804	0.82	9860946
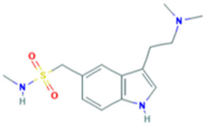	295.3988	-0.4	5358
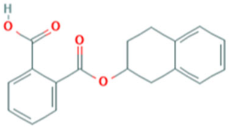	296.322	0.09	346516
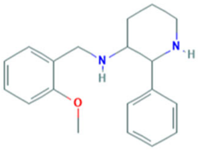	296.4114	0.87	18435769
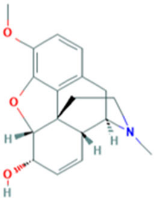	299.3688	0.45	5284371
	299.7967	0.9	4168

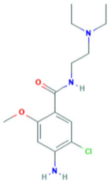			
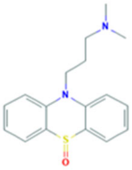	300.4178	-0.48	547559
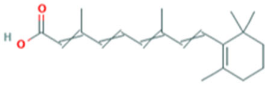	300.44	-0.49	5538
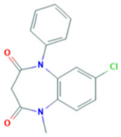	300.7409	0.36	2789
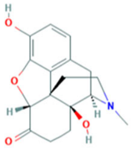	301.3414	0.35	5284604
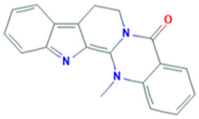	301.347	0.11	9817839
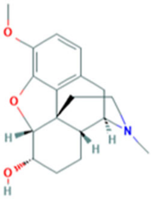	301.3846	0.55	5284543
	302.2398	-1.7	5280343

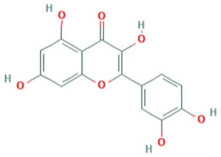			
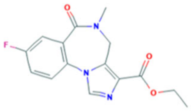	303.2919	-0.29	3373
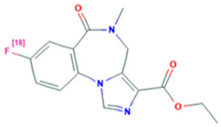	303.2919	-0.095	11652375
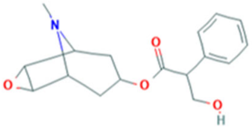	303.3572	0.23	5184
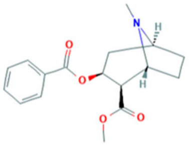	303.3572	0.6	446220
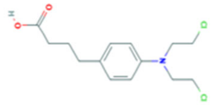	304.2096	-1.7	2708
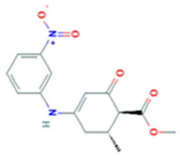	304.3018	-1	10063598
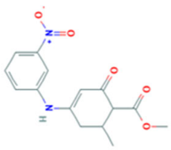	304.3018	-1	15186924

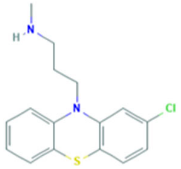	304.8337	1.38	62875
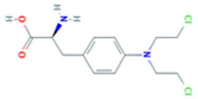	305.1974	-0.5	460612
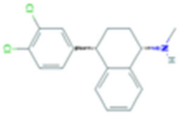	306.228	1.6	68617
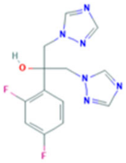	306.2734	-0.22	3365
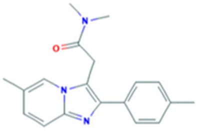	307.3944	-0.48	5732
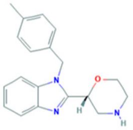	307.3944	0.36	57395287
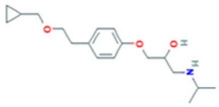	307.432	0.39	2369
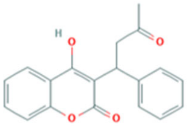	308.333	0	54678486

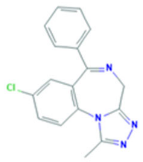	308.3792	-0.52	4781
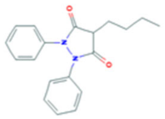	308.7665	0.02	2118
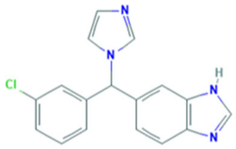	308.7665	-1	60652
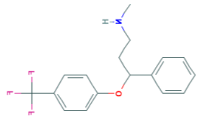	309.3293	0.72	3386
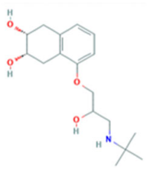	309.4046	-0.95	39147
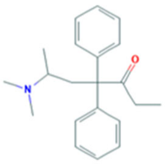	309.4504	0.9	4095
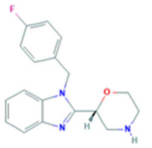	311.3577	0.77	57402315
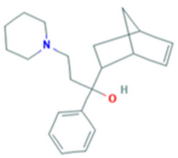	311.4662	0.85	2381

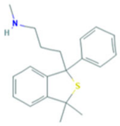	311.4842	0.22	33014
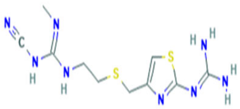	312.41	-0.82	50287
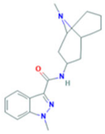	312.4138	-0.69	3510
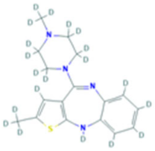	312.4318	0.78	25144104
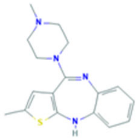	312.4318	0.78	135398745
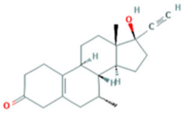	312.451	0.4	444008
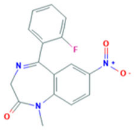	313.2871	0.07	3380
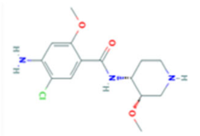	313.7803	-0.22	216236

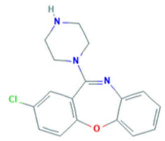	313.7829	0.6	2170
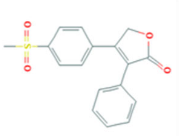	314.3552	-0.1	5090
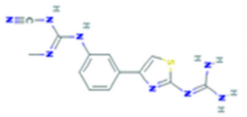	314.3672	-1.54	14022519
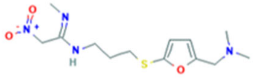	314.4018	-1.23	54429646
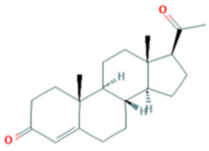	314.4668	-0.1	5994
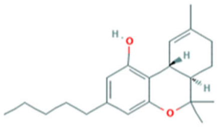	314.4668	0	16078
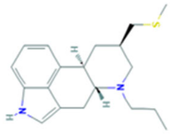	314.4878	0.3	47811
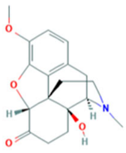	315.3682	0.57	5284603

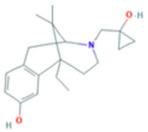	315.4546	0.6	1223
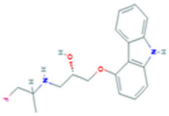	316.3741	0.58	10358392
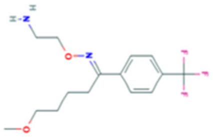	318.3371	0.79	5324346
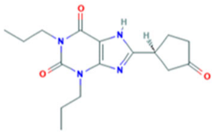	318.3748	-1.4	60949
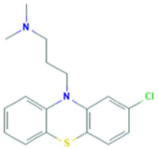	318.8605	1.02	2726
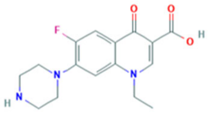	319.3345	-1.15	4539
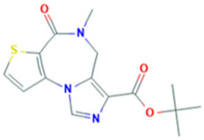	319.3776	-0.25	127382

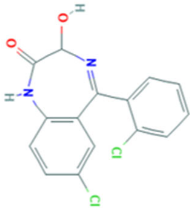	321.1562	0.44	3958
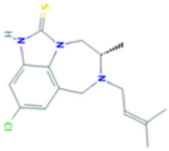	321.8641	0.28	3000237
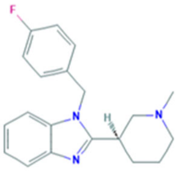	323.4119	0.43	46888857
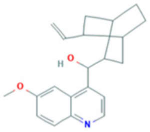	323.434	0.1	5009
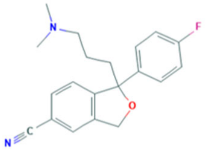	324.3967	0.99	2771
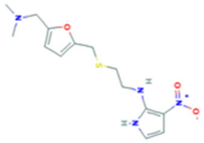	324.397	-1.12	13646638
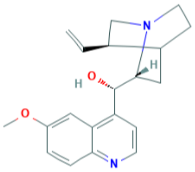	324.4218	-0.21	5953

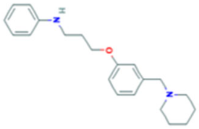	324.465	0.69	10245719
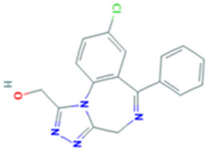	324.7659	-1.28	162244
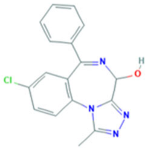	324.7659	-1.48	182017
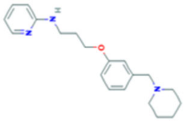	325.4528	0.69	10019237
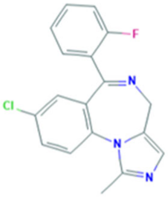	325.7688	0.37	4192
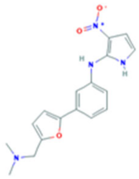	326.3542	-0.27	14022491
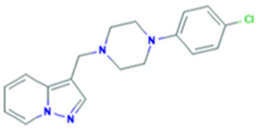	326.8249	0.64	10404144

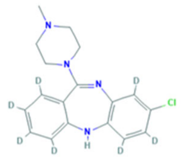	326.8249	1.3	46842852
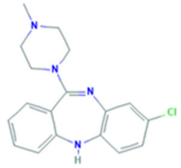	326.8249	0.7	135398737
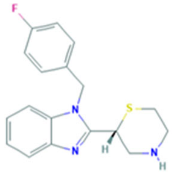	327.4183	0.89	57397056
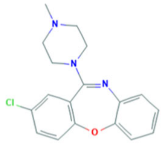	327.8097	0.8	3964
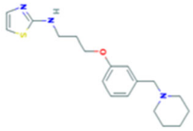	331.475	0.44	14022497
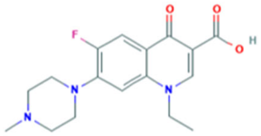	333.3613	-0.16	51081
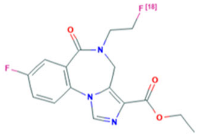	335.3088	-0.14	126761

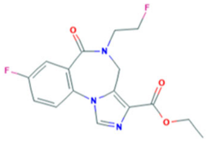	335.3088	-0.14	6426759
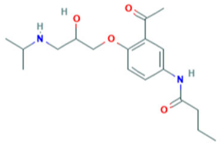	336.4302	-0.15	1978
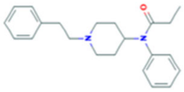	336.476	0.59	3345
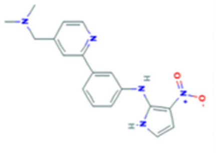	337.3804	-0.28	14022486
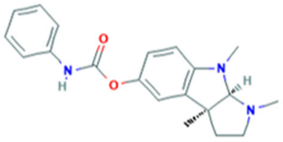	337.4206	1	192706
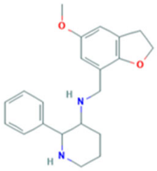	338.4486	0.39	44213642
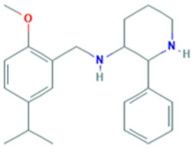	338.4918	0.92	9902443

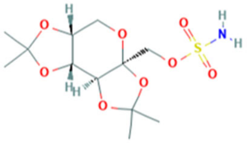	339.3598	-0.07	5284627
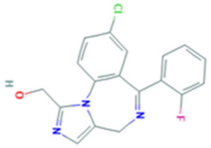	341.7682	-0.08	107917
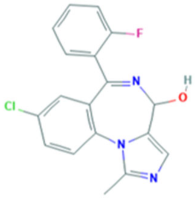	341.7682	-0.22	124449
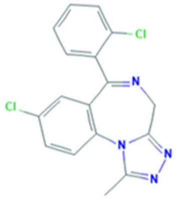	343.2086	0.67	5556
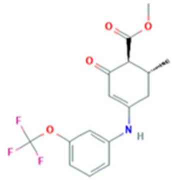	343.3007	-0.17	10088796
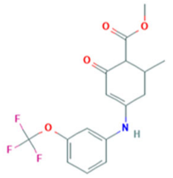	343.3007	-0.17	15186911
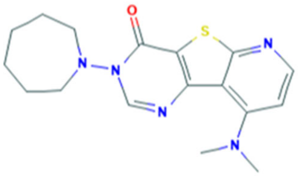	343.4458	0.43	11559235
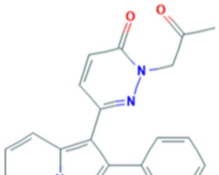			

	344.372	-0.31	11110698
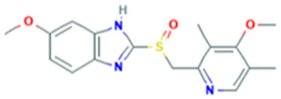	345.4154	-0.82	4594
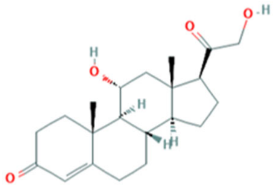	346.4656	-0.5	256277
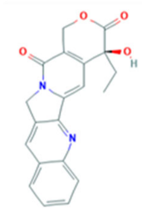	348.3574	-0.29	24360
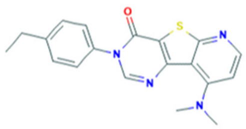	350.4374	-0.47	11530404
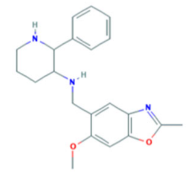	351.4474	0.49	22620080
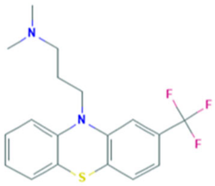	352.4155	1.44	5568

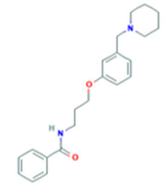	352.4754	-0.24	10498206
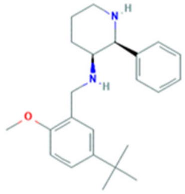	352.5186	0.98	44433393
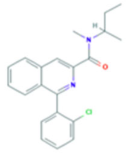	352.8597	0.48	1345
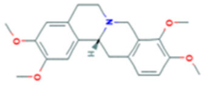	355.4328	-0.07	969488
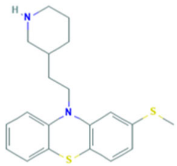	356.543	0.76	114376
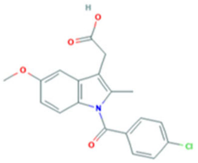	357.7897	-1.26	3715
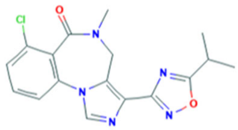	357.7957	-0.3	129710

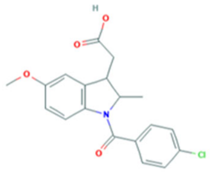	359.8055	-1.26	53394319
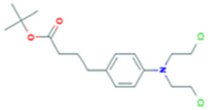	360.3168	1	83909
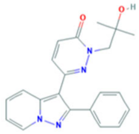	360.4146	-0.23	10937291
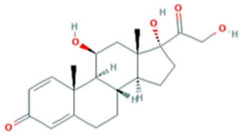	360.4492	-0.18	5755
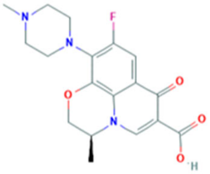	361.3717	-0.76	149096
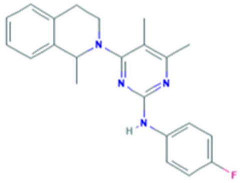	362.4485	0.68	204104
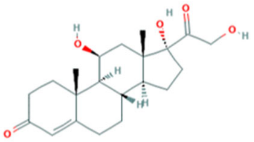	362.465	-0.22	5754

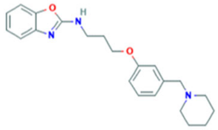	365.4742	0.22	14022499
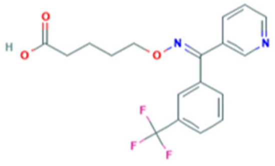	366.3379	-1	5362391
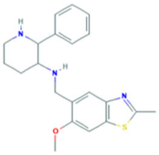	367.508	0.48	22620050
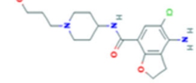	367.8717	-0.1	3052762
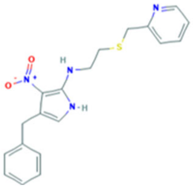	368.4526	-0.12	14022483
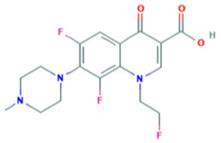	369.3415	-0.43	3357
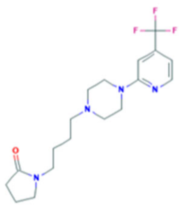	370.4157	0.16	9907401

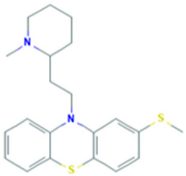	370.5698	0.26	5452
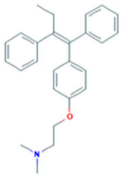	371.5212	0.92	2733526
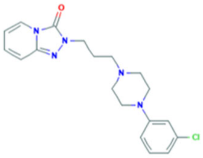	371.8657	0.3	5533
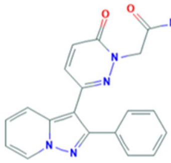	373.4134	-1	11003252
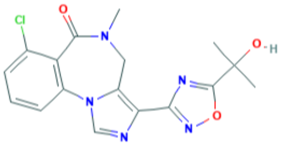	373.7951	-1.34	10384745
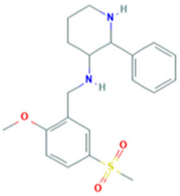	374.497	-0.15	19696721
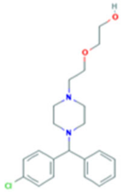	374.9065	0.36	3658

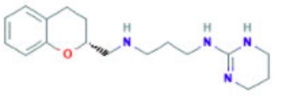	375.3344	-0.01	9907628
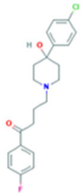	375.8662	1.33	3559
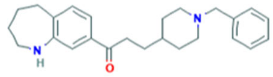	376.5406	1.14	198752
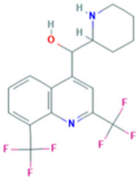	378.3142	0.63	4046
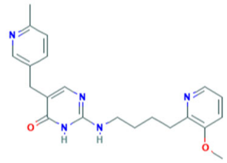	379.4608	-2	72108
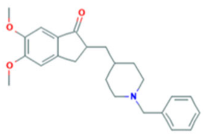	379.498	0.89	3152
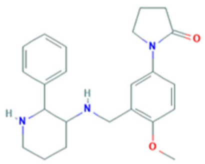	379.501	-0.22	22620062

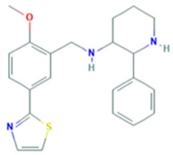	379.519	0.36	9907851
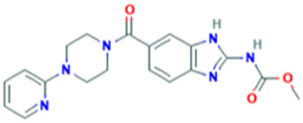	380.4054	-1.4	9864646
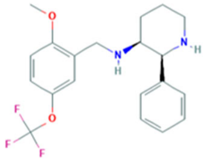	380.4079	1.11	9821217
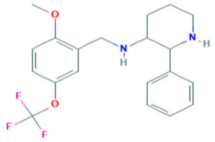	380.4079	0.63	9864647
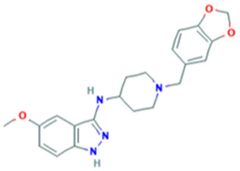	380.4456	0.57	11531010
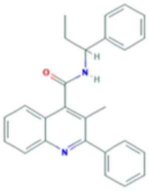	380.4884	0.3	3946663
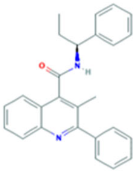	380.4884	0.3	6604009

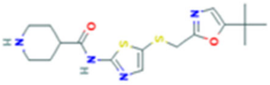	380.5222	-0.01	3025986
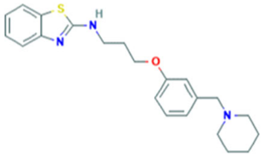	381.5348	0.14	91769
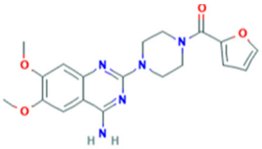	383.406	-0.9	4893
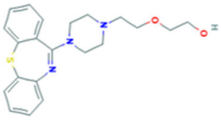	383.5074	0.7	5002
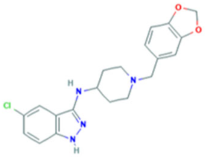	384.8615	0.83	11653882
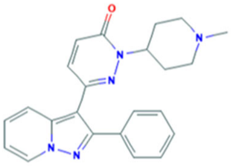	385.4676	0.06	9821511
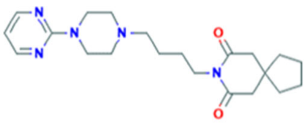	385.5082	0.49	2477

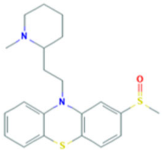	386.5692	-0.28	4078
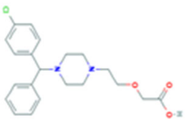	388.8901	-2.15	2678
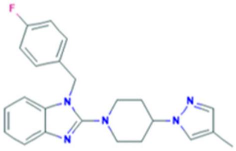	389.4741	0.32	45272895
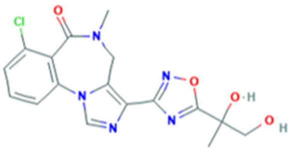	389.7945	-1.82	10091748
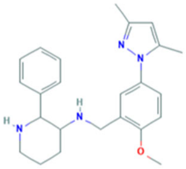	390.5272	0.11	22620075
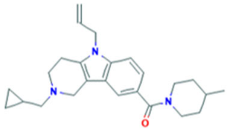	391.5552	-0.13	11502034
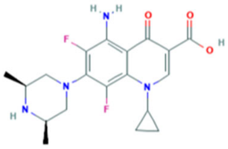	392.4038	0.41	60464

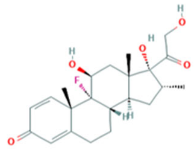	392.4661	-0.15	5743
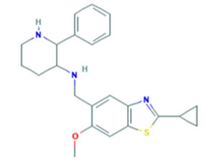	393.5458	0.62	22620085
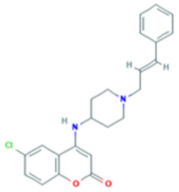	394.8969	0.13	11545677
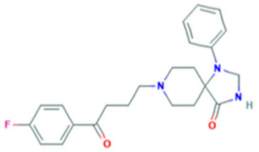	395.4753	0.26	5265
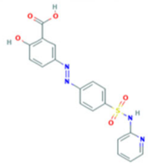	398.3924	-2.69	5339
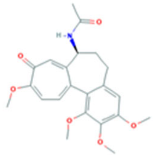	399.4426	-0.8	6167
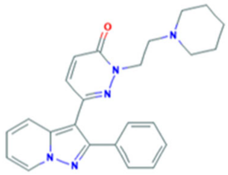	399.4944	0.38	10884606

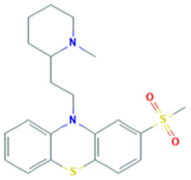	402.5686	0.18	31765
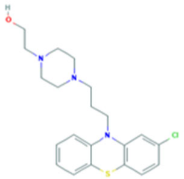	403.9659	0.7	4748
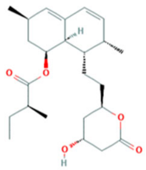	404.5454	0.85	53232
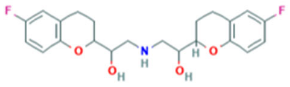	405.4398	0.57	71301
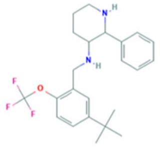	406.4889	0.96	9909299
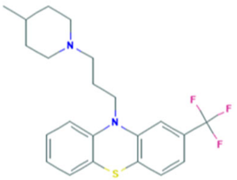	406.5069	1.44	21440942
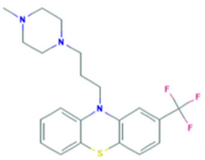	407.4947	1.43	5566

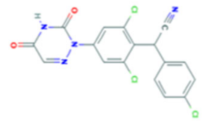	407.6337	-1.3	456389
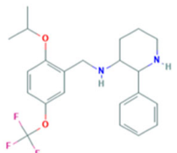	408.4615	0.41	9866153
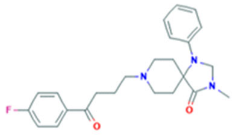	409.5021	0.46	119146
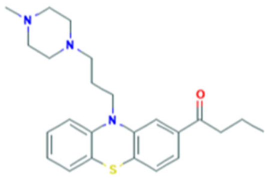	409.5884	0.83	12598
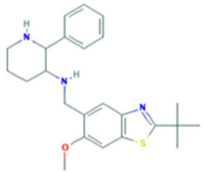	409.5884	0.85	22620091
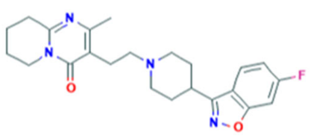	410.4899	-0.02	5073
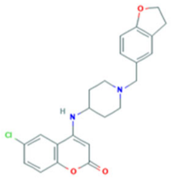	410.8963	-0.14	11531660

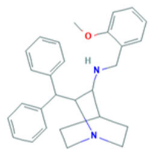	412.5736	0.37	9931510
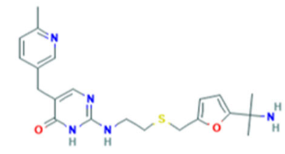	413.5366	-1.06	51671
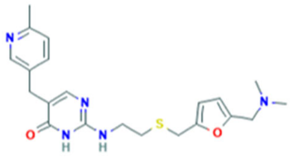	413.5366	-1.06	4375468
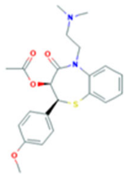	414.5184	0.3	39186
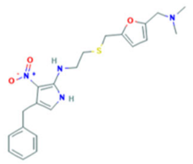	414.5214	-0.73	14022484
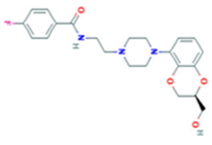	415.4631	-0.45	57347
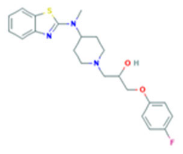	415.5243	0.78	59823

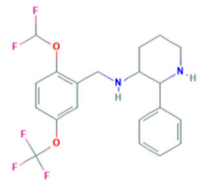	416.3881	0.88	10201984
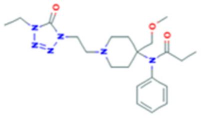	416.5222	-0.74	51263
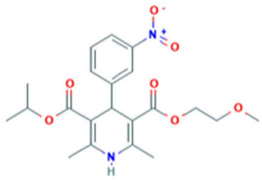	418.4456	0.08	4497
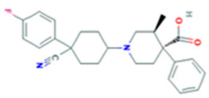	420.5253	-0.7	54385
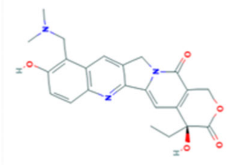	421.4518	-0.42	60700
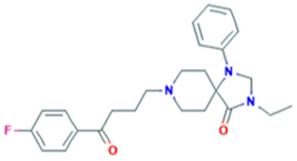	423.5289	-0.01	13497176
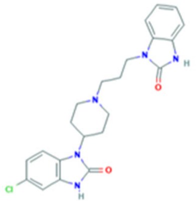	425.9139	-0.85	3151

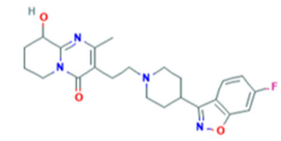	426.4893	-0.67	115237
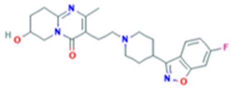	426.4893	-0.67	475100
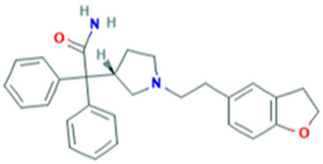	426.5572	-0.62	444031
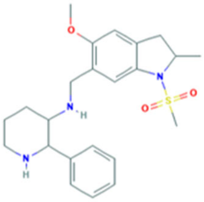	429.5762	-0.42	19696654
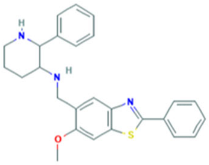	429.5788	1.26	22620066
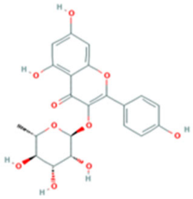	432.383	0.548	5316673
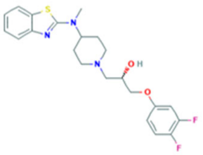	433.5144	0.59	65998

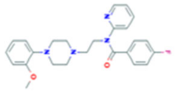	434.5119	0.09	6603915
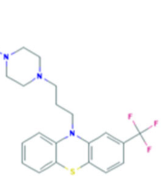	437.5209	1.51	3372
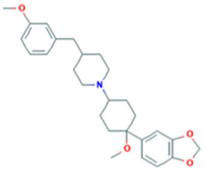	437.5778	0.74	10388384
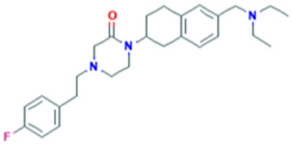	437.5989	0.48	44592215
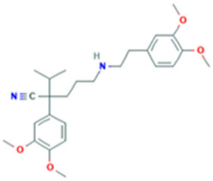	440.5814	-0.64	104972
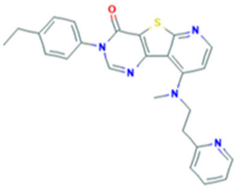	441.5496	-0.8	44416780
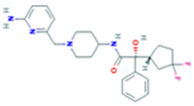	444.5226	-0.89	9846311

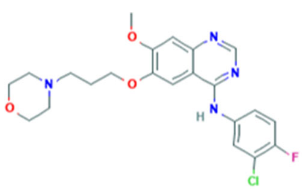	446.9046	-0.5	123631
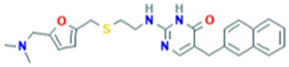	448.5818	-1.3	14022522
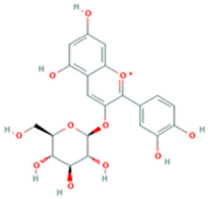	449.3903	0.15	441667
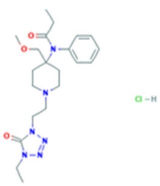	452.9801	-0.82	64761
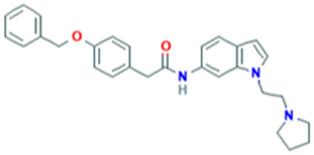	453.5828	-0.59	70691333
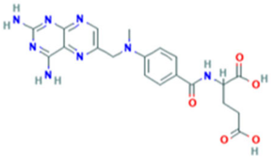	454.4444	-1.51	4112
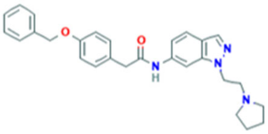	454.5706	-0.2	11775399

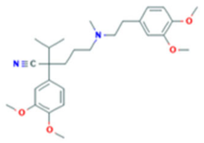	454.6082	-0.64	2520
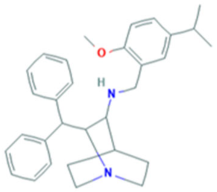	454.654	0.48	6426143
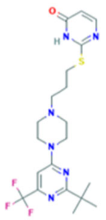	456.5269	0.3	9916104
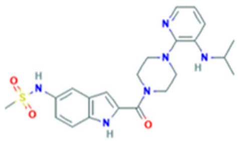	456.5616	-1.52	5625
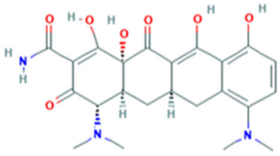	457.4822	-0.52	54675783
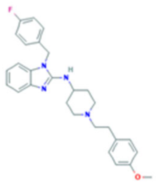	458.5771	1.1	2247
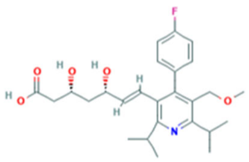	459.5563	-1.3	446156

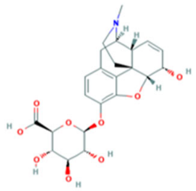	461.4676	-2.15	5484731
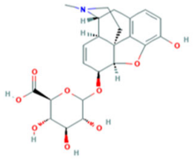	461.4676	-2.09	9847115
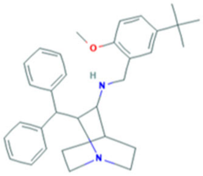	468.6808	0.46	17905241
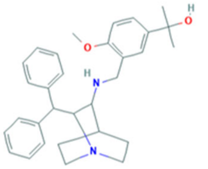	470.6534	-0.89	6426129
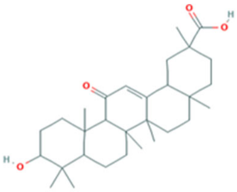	470.691	-1.4	3230
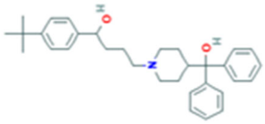	471.6814	0.64	5405
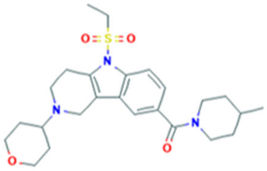	473.6292	-1.15	24894028

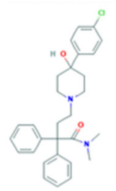	477.0419	0.77	3955
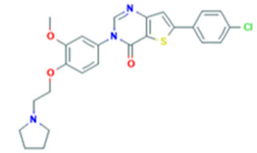	481.9929	0.78	9826520
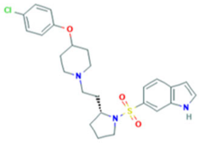	488.0403	-0.04	10028436
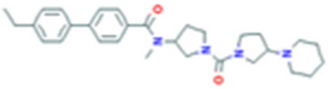	488.6716	0.81	73226213
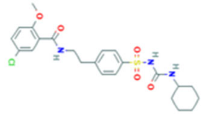	494.0013	-0.9	3488
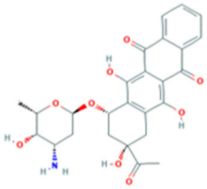	497.5006	0	42890
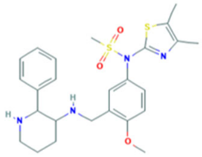	500.6728	-0.37	19696689

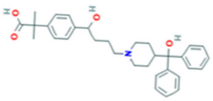	501.6644	-0.98	3348
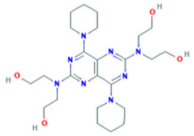	504.6312	0	3108
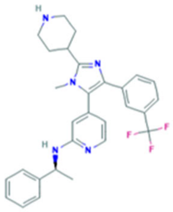	505.5835	0.01	9806182
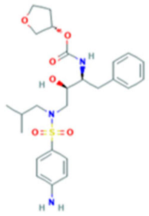	505.628	-0.56	65016
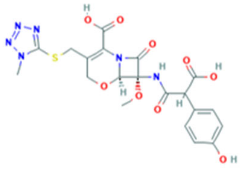	520.4728	-2.52	47499
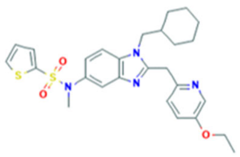	524.6948	-1.15	57390958
	531.4316	-0.63	3823

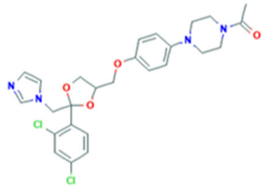			
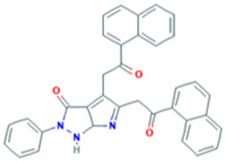	535.6008	0.02	343473
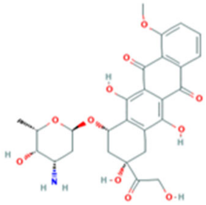	543.5262	-0.83	31703
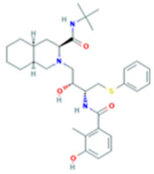	567.7852	-0.93	64143
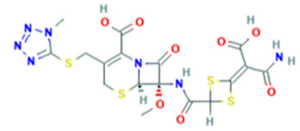	575.6034	-1.89	53025
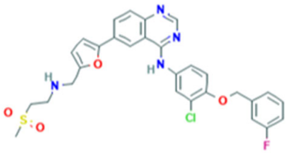	581.0568	-0.03	208908
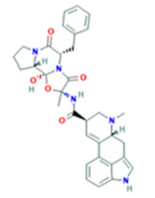	581.67	-1.1	8223

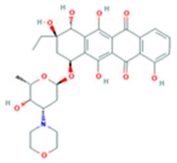	585.6066	0.56	6917994
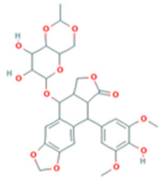	588.564	-2	3310

Figure S1. Linear diagram showing correlation between the experimental and calculated LogBB values of the Subset 1 molecules in the training (black) and the test set (red).

Fold #1

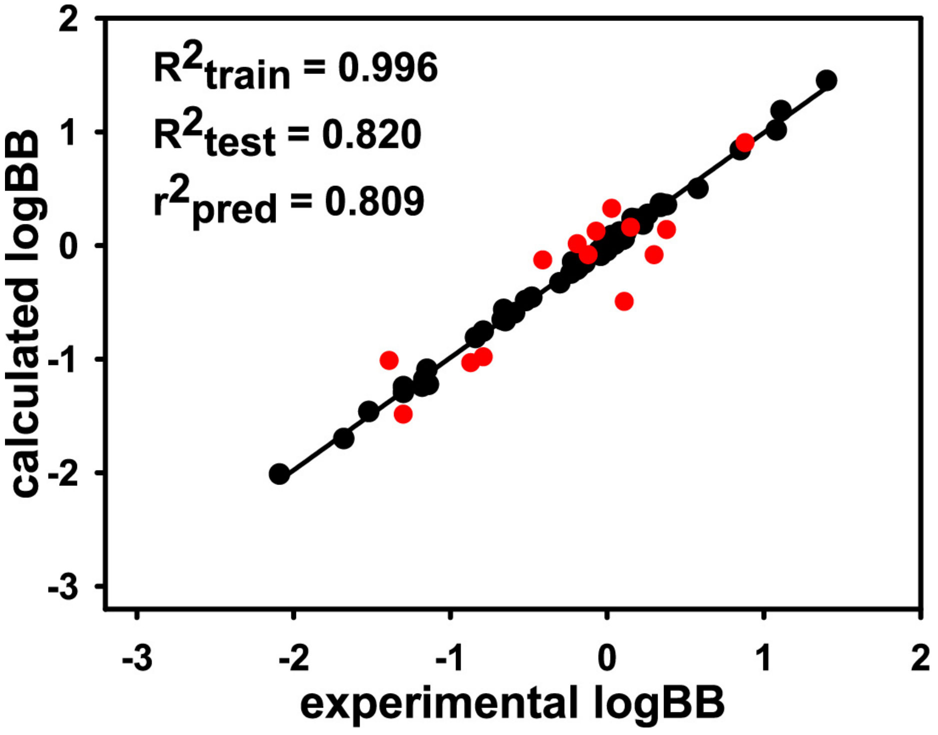

Compounds in the training set: 444, 1207, 2022, 2083, 2119, 2164, 2284, 2554, 2578, 2803, 3241, 3394, 3672, 3676, 4058, 4064, 4421, 4737, 4763, 4828, 4914, 5064, 5155, 5193, 5838, 5842, 6009, 6468, 6473, 8942, 18047, 24066, 25255, 31423, 33039, 56051, 65860, 66994, 72138, 90274, 94957, 156391, 451819, 1497794, 3034183, 3763607, 6604200, 10060538, 10377120, 10444765, 11777724, 12889418, 13071367, 14022509, 21675832, 23274095, 118753220.

Compounds in the test set: 1486, 2473, 3608, 4158, 4909, 5206, 6623, 50599, 77870, 3000715, 9837317, 12032949, 14840722, 44214615.

Figure S2. Linear diagram showing correlation between the experimental and calculated LogBB values of the Subset 1 molecules in the training (black) and the test set (red).

Fold #2

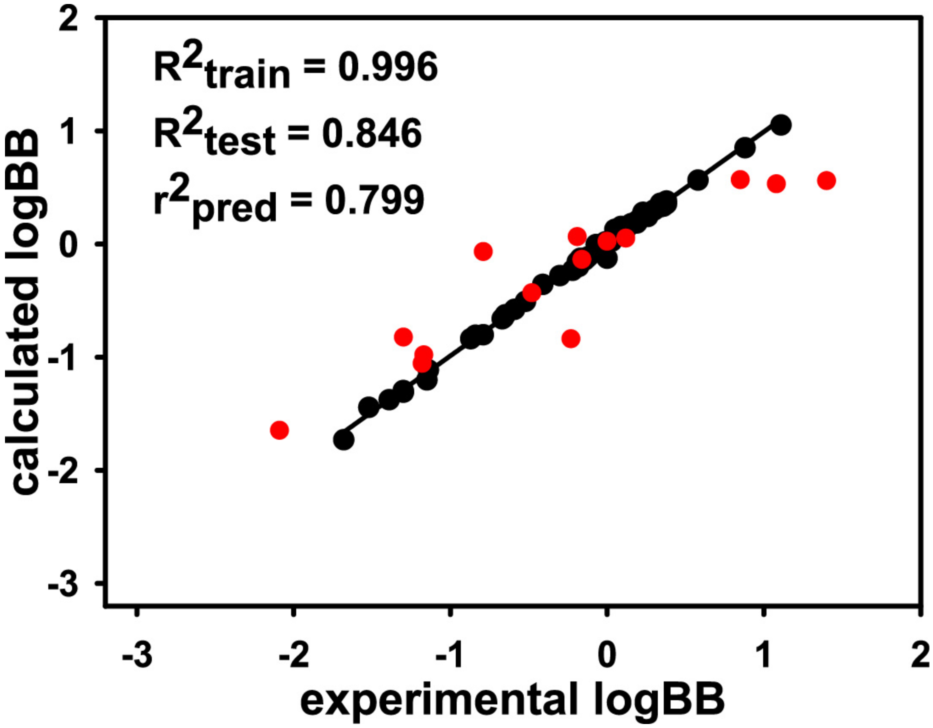

Compounds in the training set: 1207, 1486, 2022, 2083, 2164, 2284, 2473, 2554, 2578, 2803, 3394, 3608, 3672, 3676, 4064, 4158, 4421, 4737, 4828, 4909, 4914, 5064, 5193, 5206, 5838, 5842, 6468, 6473, 6623, 8942, 18047, 25255, 31423, 33039, 50599, 56051, 66994, 72138, 77870, 90274, 94957, 156391, 1497794, 3000715, 3034183, 3763607, 9837317, 10060538, 10377120, 10444765, 11777724, 12032949, 13071367, 14022509, 14840722, 23274095.

Compounds in the test set: 444, 2119, 3241, 4058, 4763, 5155, 6009, 24066, 65860, 451819, 6604200, 12889418, 21675832, 118753220.

Figure S3. Linear diagram showing correlation between the experimental and calculated LogBB values of the Subset 1 molecules in the training (black) and the test set (red).

Fold #3

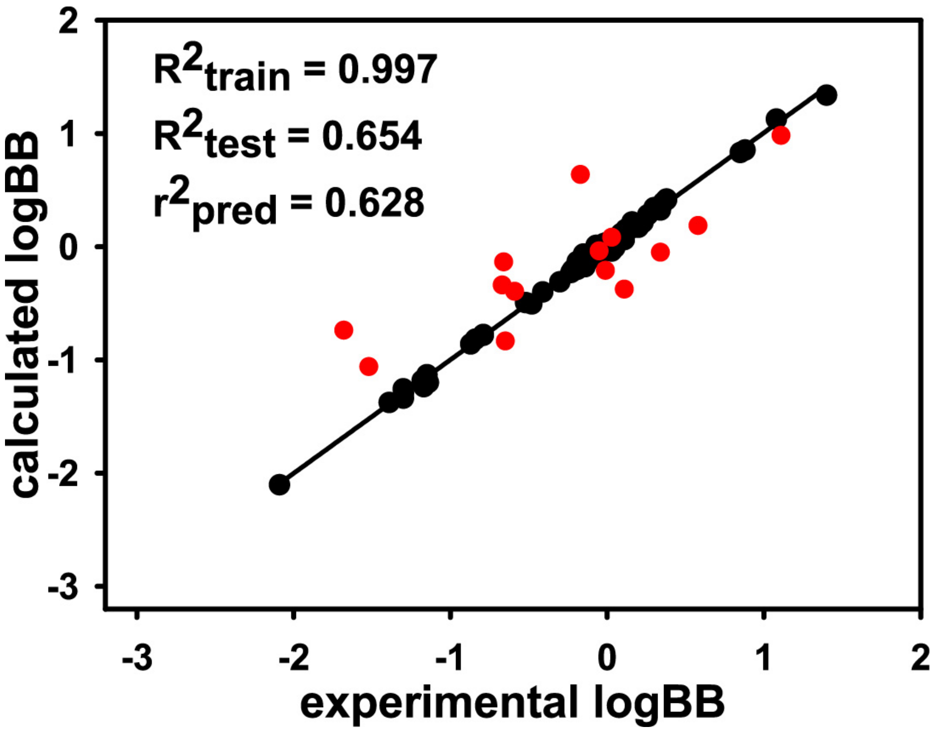

Compounds in the training set: 444, 1486, 2022, 2083, 2119, 2164, 2473, 2554, 2578, 2803, 3241, 3608, 3672, 3676, 4058, 4064, 4158, 4737, 4763, 4828, 4909, 4914, 5155, 5193, 5206, 5838, 5842, 6009, 6473, 6623, 8942, 24066, 25255, 31423, 50599, 56051, 65860, 66994, 77870, 90274, 156391, 451819, 1497794, 3000715, 3763607, 6604200, 9837317, 10060538, 10444765, 11777724, 12032949, 12889418, 14022509, 14840722, 21675832, 44214615, 118753220.

Compounds in the test set: 1207, 2284, 3394, 4421, 5064, 6468, 18047, 33039, 72138, 94957, 3034183, 10377120, 13071367, 23274095.

Figure S4. Linear diagram showing correlation between the experimental and calculated LogBB values of the Subset 2 molecules in the training (black) and the test set (red).

Fold #1

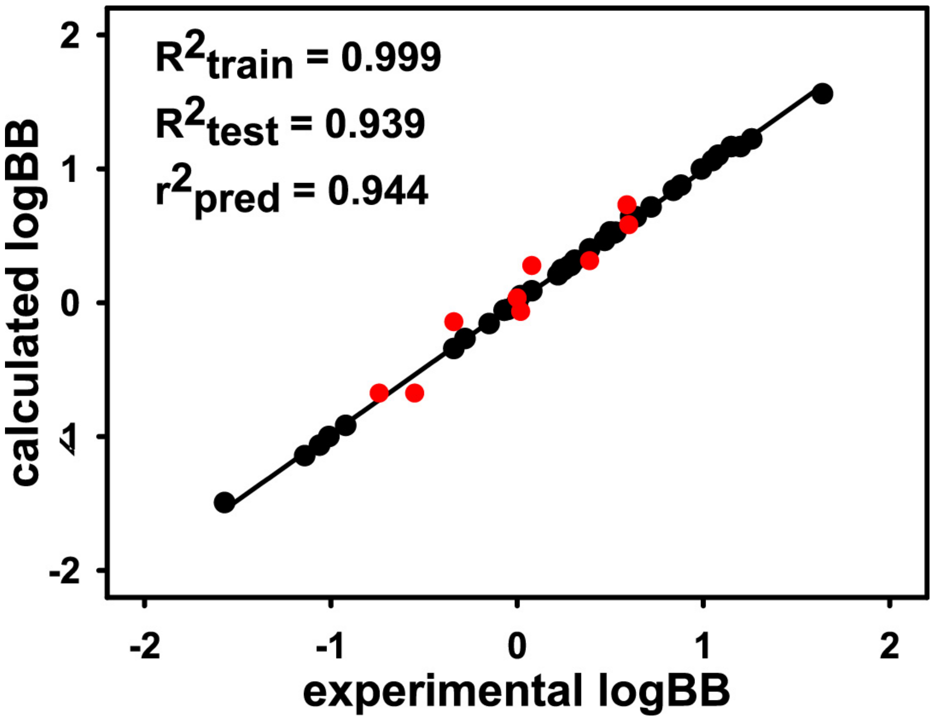

Compounds in the training set: 1775, 2249, 2756, 2995, 2997, 3100, 3162, 3878, 3918, 4171, 4184, 4205, 4463, 4946, 5092, 5095, 5253, 5505, 6086, 9034, 33741, 37614, 43473, 77991, 159642, 3034012, 5281708, 5359272, 6916184, 9796408, 10470115, 13755681, 14022511, 14577084, 14590445, 15524033, 19427054, 44383796, 70517986.

Compounds in the test set: 2555, 4631, 5726, 5983, 28315, 443371, 9861160, 44568616, 135458393.

Figure S5. Linear diagram showing correlation between the experimental and calculated LogBB values of the Subset 2 molecules in the training (black) and the test set (red).

Fold #2

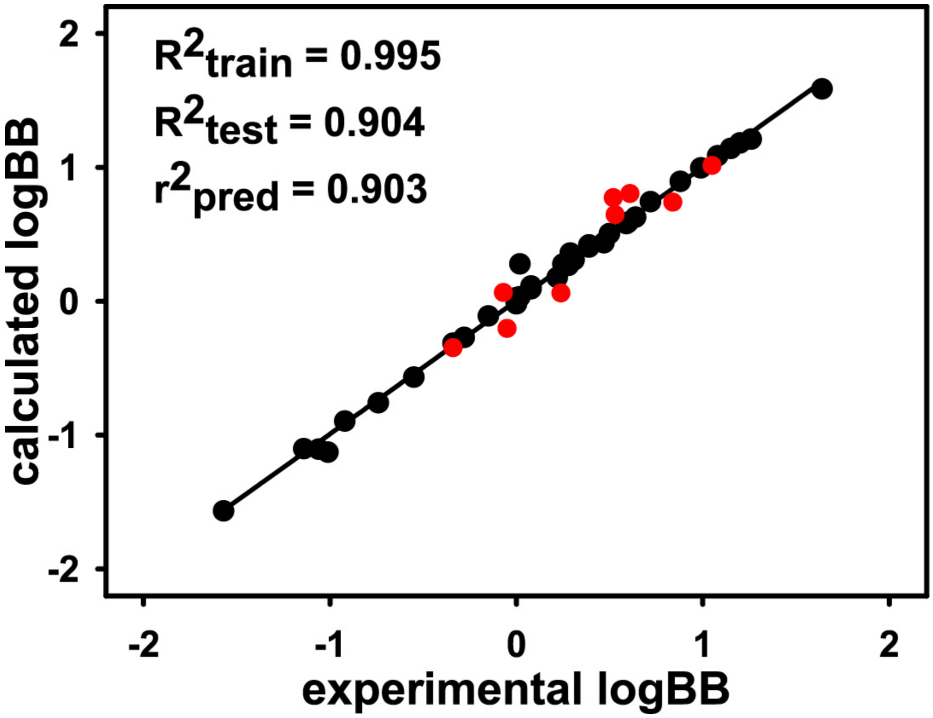

Compounds in the training set: 2249, 2555, 2756, 2995, 3100, 3162, 3878, 3918, 4171, 4184, 4463, 4631, 5095, 5253, 5505, 5726, 5983, 6086, 28315, 33741, 37614, 43473, 77991, 443371, 3034012, 5281708, 5359272, 6916184, 9796408, 9861160, 10470115, 14022511, 14577084, 14590445, 15524033, 19427054, 44383796, 44568616, 135458393.

Compounds in the test set: 1775, 2997, 4205, 4946, 5092, 9034, 159642, 13755681, 70517986.

Figure S6. Linear diagram showing correlation between the experimental and calculated LogBB values of the Subset 2 molecules in the training (black) and the test set (red).

Fold #3

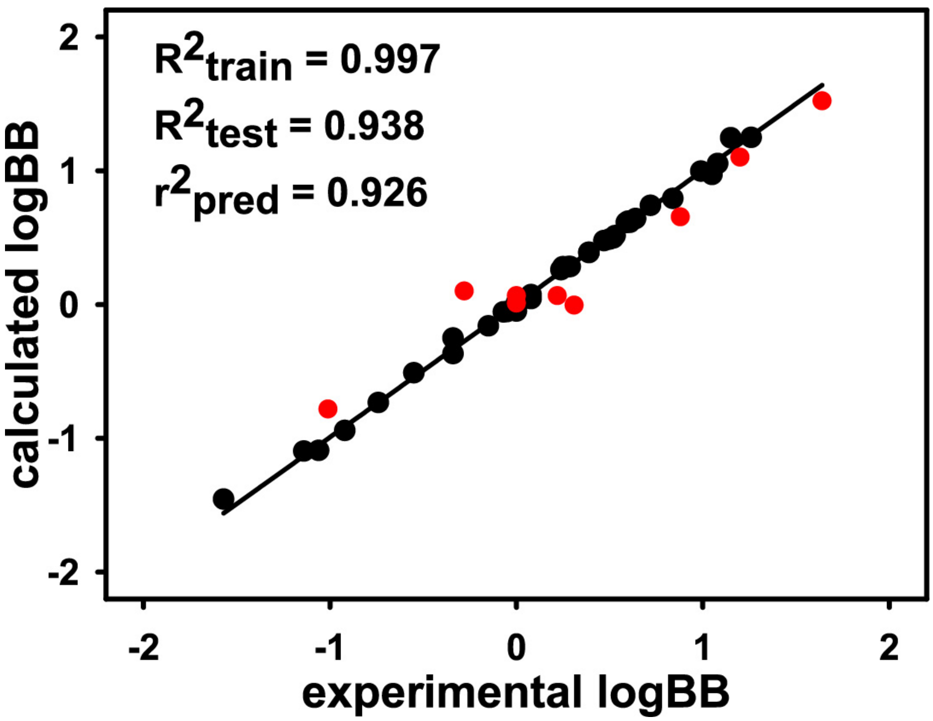

Compounds in the training set: 1775, 2249, 2555, 2756, 2995, 2997, 3100, 3162, 3878, 3918, 4171, 4184, 4205, 4463, 4631, 4946, 5092, 5095, 5726, 5983, 9034, 28315, 33741, 37614, 43473, 159642, 443371, 3034012, 5281708, 9861160, 10470115, 13755681, 14022511, 14577084, 14590445, 44383796, 44568616, 70517986, 135458393.

Compounds in the test set: 5253, 5505, 6086, 77991, 5359272, 6916184, 9796408, 15524033, 19427054.

Figure S7. Linear diagram showing correlation between the experimental and calculated LogBB values of the Subset 3 molecules in the training (black) and the test set (red).

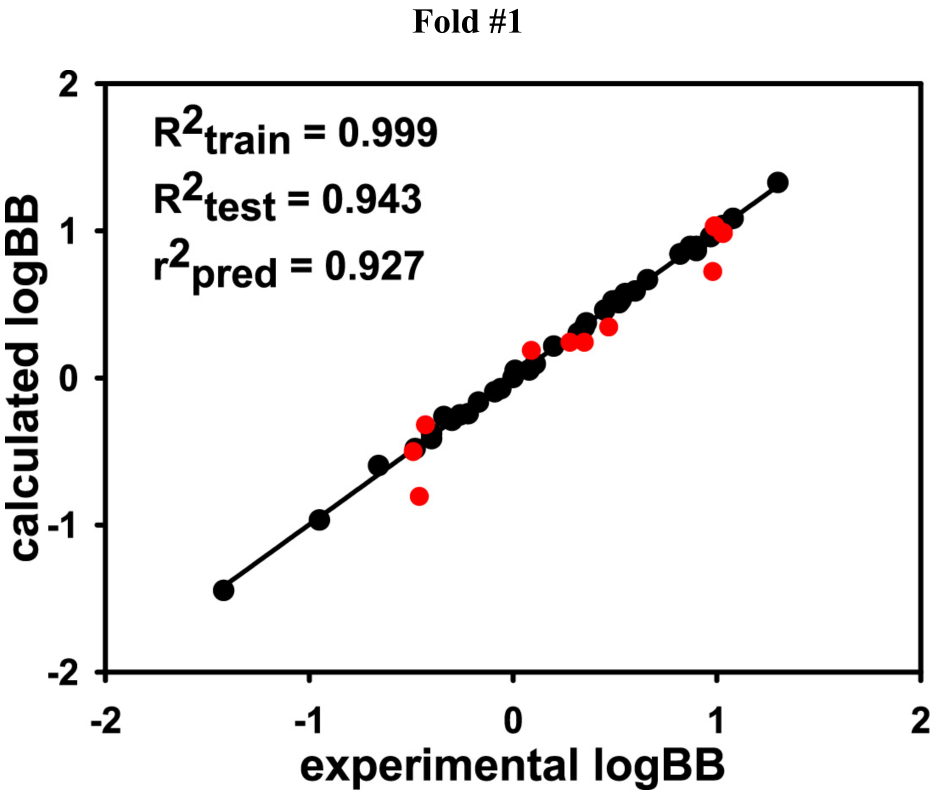

Compounds in the training set: 2160, 2583, 2789, 3158, 3308, 4011, 4168, 4616, 4736, 4911, 4926, 4992, 5358, 5402, 6013, 9651, 67101, 68778, 74981, 121249, 166560, 174174, 197322, 441300, 547559, 3035905, 5284371, 5284543, 5284604, 5288826, 9817839, 9839073, 9860946, 9903970, 10265481, 14022481, 18435769, 22154175, 29939457, 44568615.

Compounds in the test set: 3016, 3696, 4595, 5538, 5584, 89657, 133741, 163091, 346516, 9971484.

Figure S8. Linear diagram showing correlation between the experimental and calculated LogBB values of the Subset 3 molecules in the training (black) and the test set (red).

Fold #2

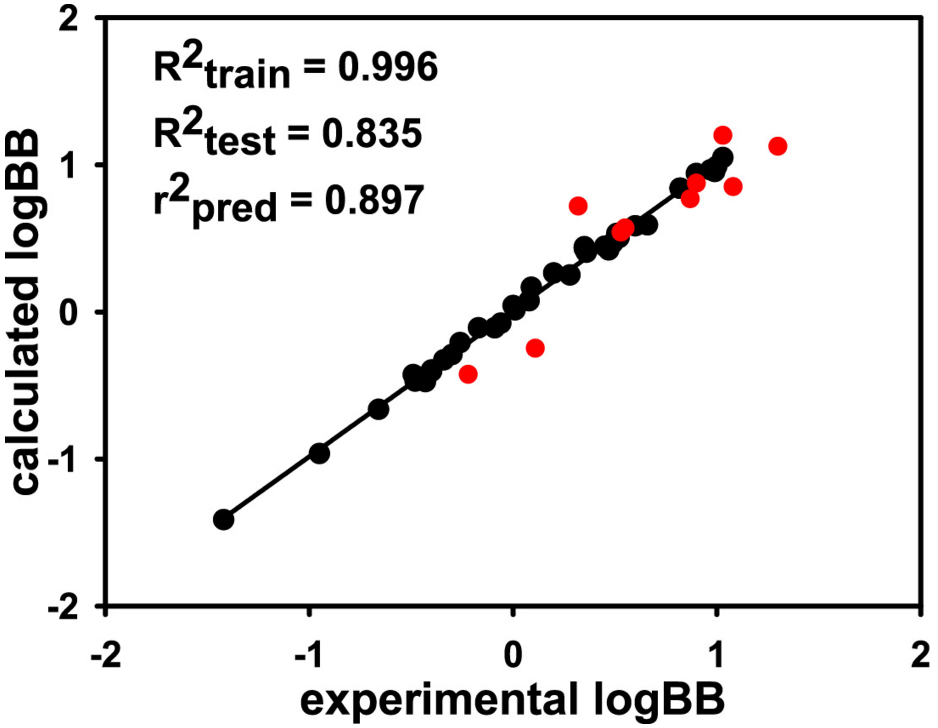

Compounds in the training set: 2583, 2789, 3016, 3158, 3308, 3696, 4168, 4595, 4616, 4736, 4911, 4992, 5358, 5402, 5538, 5584, 6013, 67101, 68778, 74981, 89657, 133741, 163091, 166560, 174174, 197322, 346516, 547559, 3035905, 5284371, 5284604, 5288826, 9839073, 9860946, 9971484, 10265481, 14022481, 22154175, 29939457, 44568615.

Compounds in the test set: 2160, 4011, 4926, 9651, 121249, 441300, 5284543, 9817839, 9903970, 18435769.

Figure S9. Linear diagram showing correlation between the experimental and calculated LogBB values of the Subset 3 molecules in the training (black) and the test set (red).

Fold #3

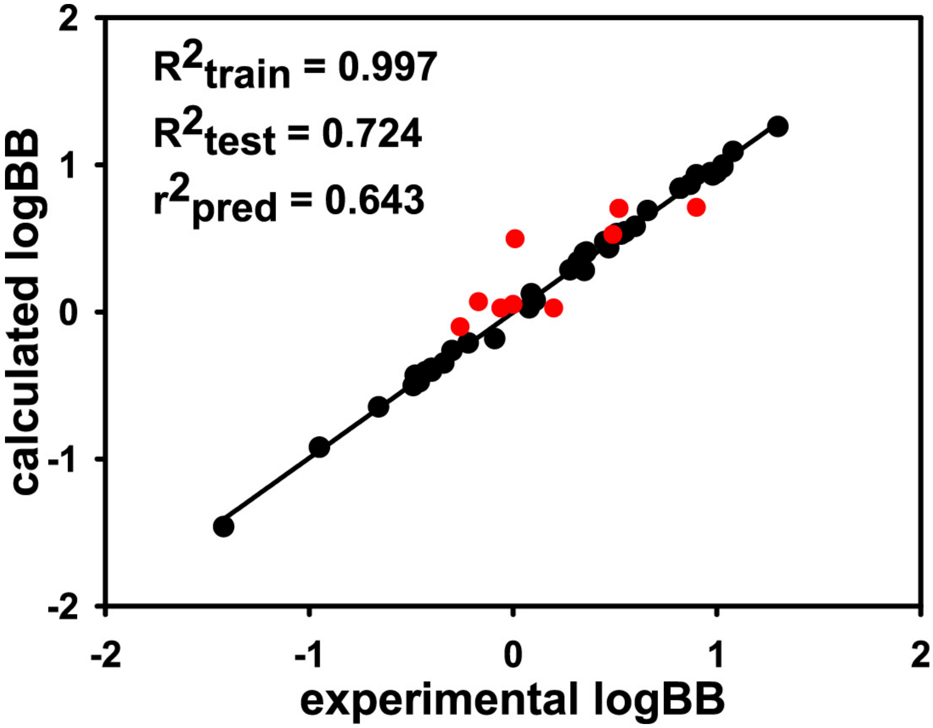

Compounds in the training set: 2160, 2789, 3016, 3158, 3308, 3696, 4011, 4595, 4616, 4736, 4911, 4926, 5358, 5402, 5538, 5584, 9651, 67101, 68778, 74981, 89657, 121249, 133741, 163091, 166560, 197322, 346516, 441300, 547559, 5284371, 5284543, 5284604, 9817839, 9839073, 9860946, 9903970, 9971484, 14022481, 18435769, 29939457.

Compounds in the test set: 2583, 4168, 4992, 6013, 174174, 3035905, 5288826, 10265481, 22154175, 44568615.

Figure S10. Linear diagram showing correlation between the experimental and calculated LogBB values of the Subset 4 molecules in the training (black) and the test set (red).

Fold #1

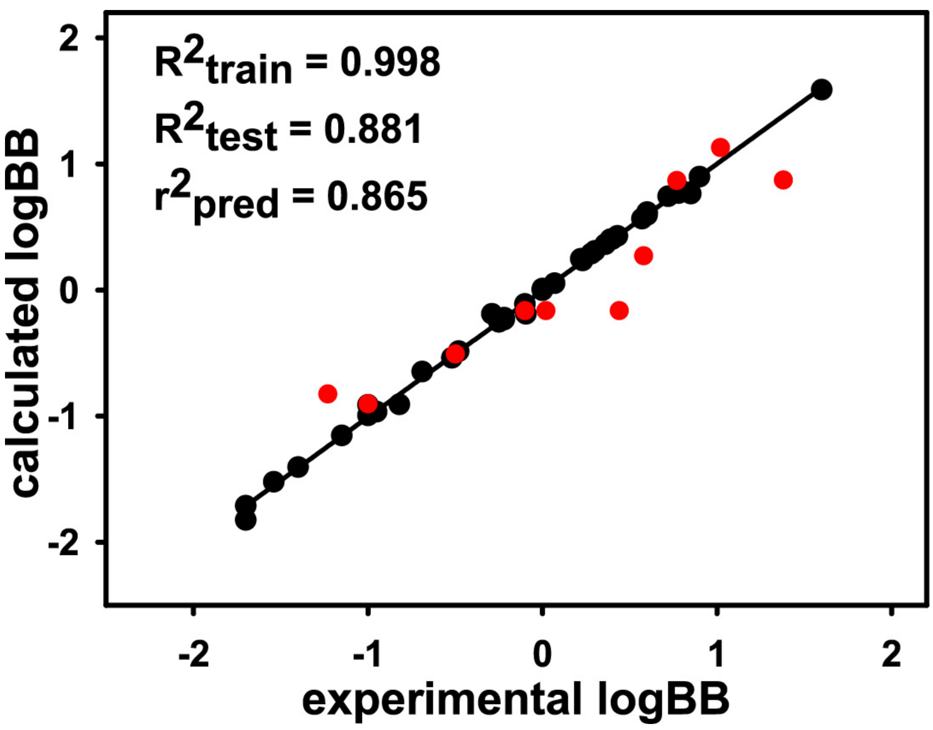

Compounds in the training set: 1223, 2170, 2369, 2381, 2708, 3365, 3373, 3380, 3386, 3510, 4095, 4539, 4781, 5090, 5184, 5732, 16078, 33014, 39147, 47811, 50287, 60652, 60949, 68617, 127382, 216236, 444008, 446220, 3000237, 5280343, 5284603, 5324346, 10063598, 11652375, 14022519, 25144104, 46888857, 54678486, 57395287, 135398745.

Compounds in the test set: 2118, 2726, 3958, 5994, 62875, 460612, 10358392, 15186924, 54429646, 57402315.

Figure S11. Linear diagram showing correlation between the experimental and calculated LogBB values of the Subset 4 molecules in the training (black) and the test set (red).

Fold #2

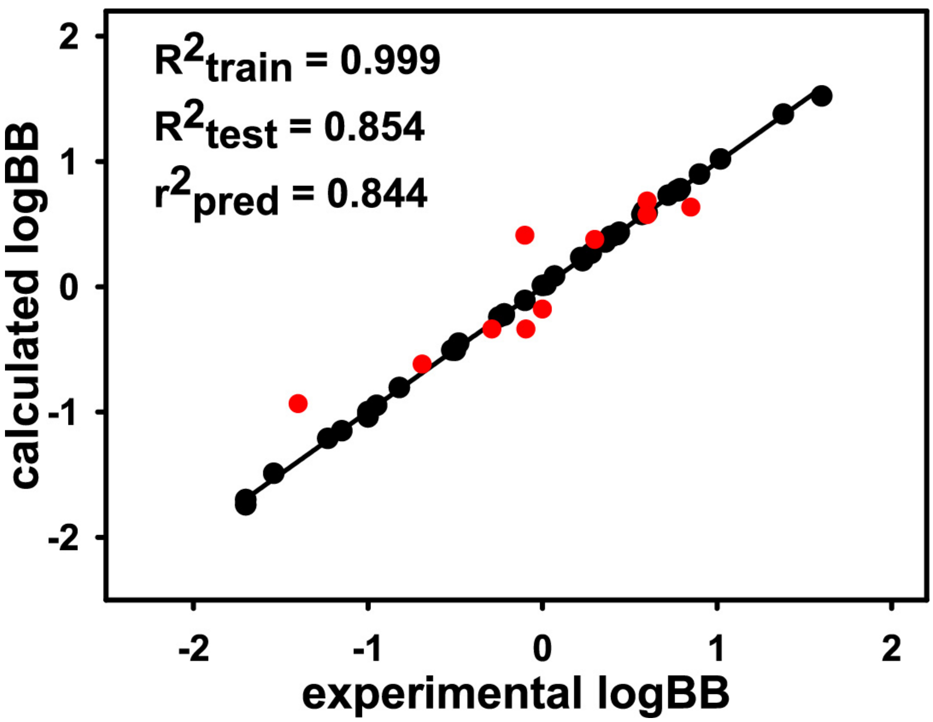

Compounds in the training set: 2170, 2369, 2708, 3365, 3380, 3386, 4095, 4539, 4781, 5184, 5732, 33014, 39147, 50287, 60652, 68617, 127382, 216236, 444008, 3000237, 5280343, 5284603, 5324346, 10063598, 14022519, 25144104, 46888857, 54678486, 57395287, 135398745, 2118, 2726, 3958, 5994, 62875, 460612, 10358392, 15186924, 54429646, 57402315.

Compounds in the test set: 1223, 2381, 3373, 3510, 5090, 16078, 47811, 60949, 446220, 11652375.

Figure S12. Linear diagram showing correlation between the experimental and calculated LogBB values of the Subset 4 molecules in the training (black) and the test set (red).

Fold #3

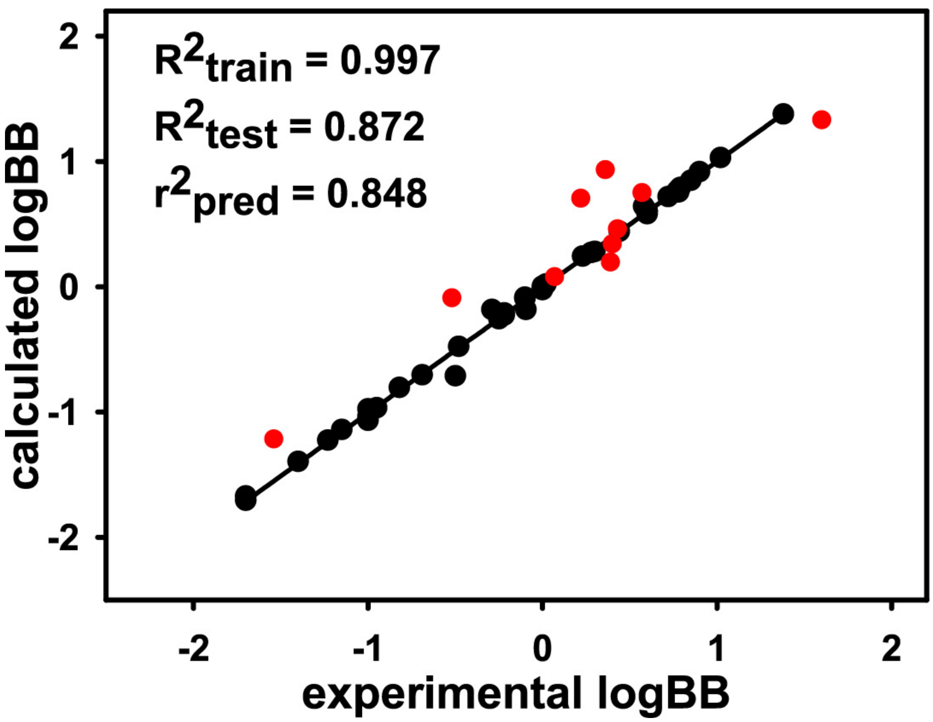

Compounds in the training set: 1223, 2118, 2170, 2381, 2708, 2726, 3365, 3373, 3386, 3510, 3958, 4095, 4539, 5090, 5184, 5732, 5994, 16078, 39147, 47811, 50287, 60652, 60949, 62875, 127382, 216236, 446220, 460612, 3000237, 5280343, 5324346, 10063598, 10358392, 11652375, 15186924, 25144104, 54429646, 54678486, 57402315, 135398745.

Compounds in the test set: 2369, 3380, 4781, 33014, 68617, 444008, 5284603, 14022519, 46888857, 57395287.

Figure S13. Linear diagram showing correlation between the experimental and calculated LogBB values of the Subset 5 molecules in the training (black) and the test set (red).

Fold #1

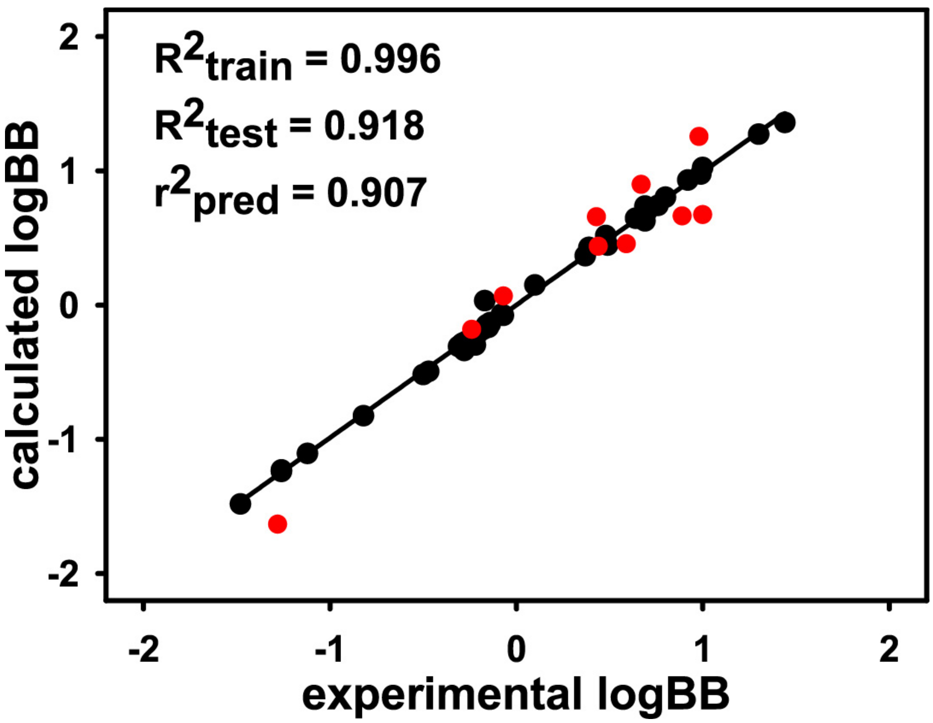

Compounds in the training set: 1345, 1978, 2771, 3715, 3964, 4192, 4594, 5009, 5568, 5755, 5953, 24360, 51081, 107917, 114376, 124449, 126761, 129710, 182017, 192706, 256277, 969488, 6426759, 9902443, 10019237, 10088796, 10245719, 10404144, 10937291, 11110698, 11530404, 13646638, 14022486, 14022491, 15186911, 22620080, 44213642, 46842852, 53394319, 135398737.

Compounds in the test set: 3345, 5556, 83909, 162244, 5284627, 10498206, 11559235, 14022497, 44433393, 57397056.

Figure S14. Linear diagram showing correlation between the experimental and calculated LogBB values of the Subset 5 molecules in the training (black) and the test set (red).

Fold #2

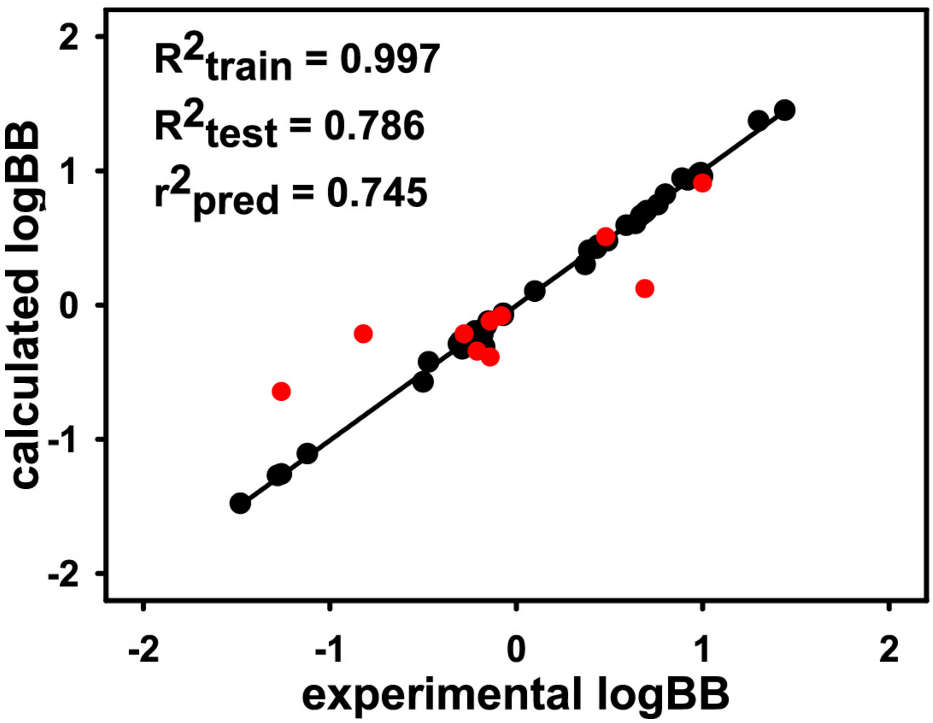

Compounds in the training set: 1978, 2771, 3345, 3964, 4192, 5009, 5556, 5568, 5755, 24360, 51081, 83909, 114376, 124449, 129710, 162244, 182017, 256277, 969488, 5284627, 9902443, 10019237, 10088796, 10404144, 10498206, 10937291, 11110698, 11530404, 11559235, 13646638, 14022491, 14022497, 15186911, 22620080, 44213642, 44433393, 46842852, 53394319, 57397056, 135398737.

Compounds in the test set: 1345, 3715, 4594, 5953, 107917, 126761, 192706, 6426759, 10245719, 14022486.

Figure S15. Linear diagram showing correlation between the experimental and calculated LogBB values of the Subset 5 molecules in the training (black) and the test set (red).

Fold #3

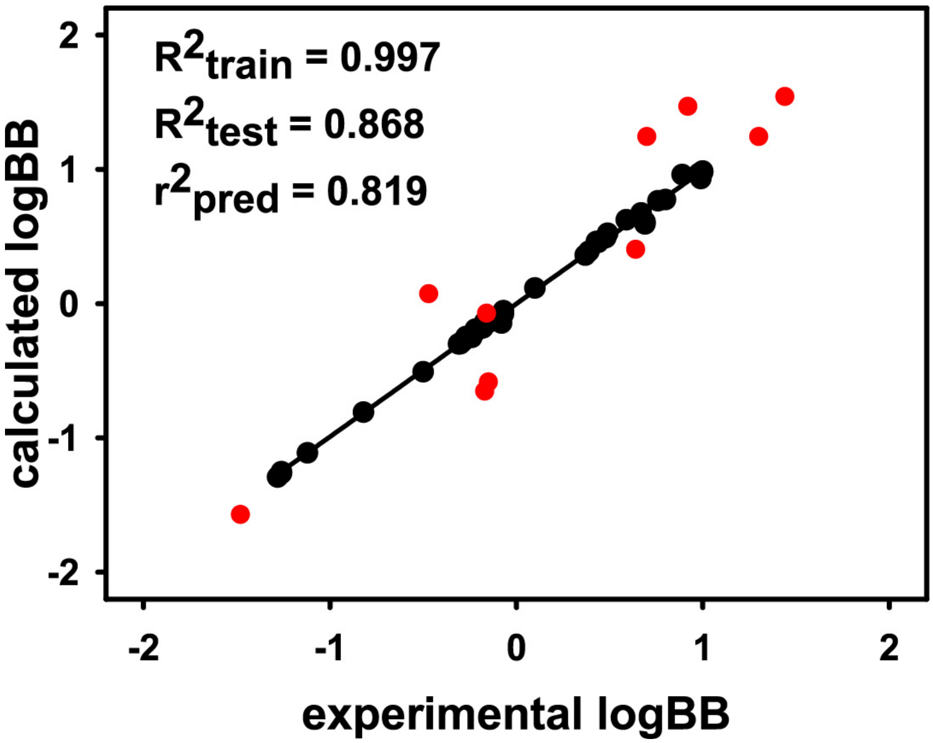

Compounds in the training set: 1345, 2771, 3345, 3715, 3964, 4192, 4594, 5009, 5556, 5755, 5953, 24360, 83909, 107917, 114376, 124449, 126761, 129710, 162244, 192706, 256277, 969488, 5284627, 6426759, 10019237, 10088796, 10245719, 10498206, 10937291, 11110698, 11559235, 13646638, 14022486, 14022491, 14022497, 22620080, 44213642, 44433393, 53394319, 57397056.

Compounds in the test set: 1978, 5568, 51081, 182017, 9902443, 10404144, 11530404, 15186911, 46842852, 135398737.

Figure S16. Linear diagram showing correlation between the experimental and calculated LogBB values of the Subset 6 molecules in the training (black) and the test set (red).

Fold #1

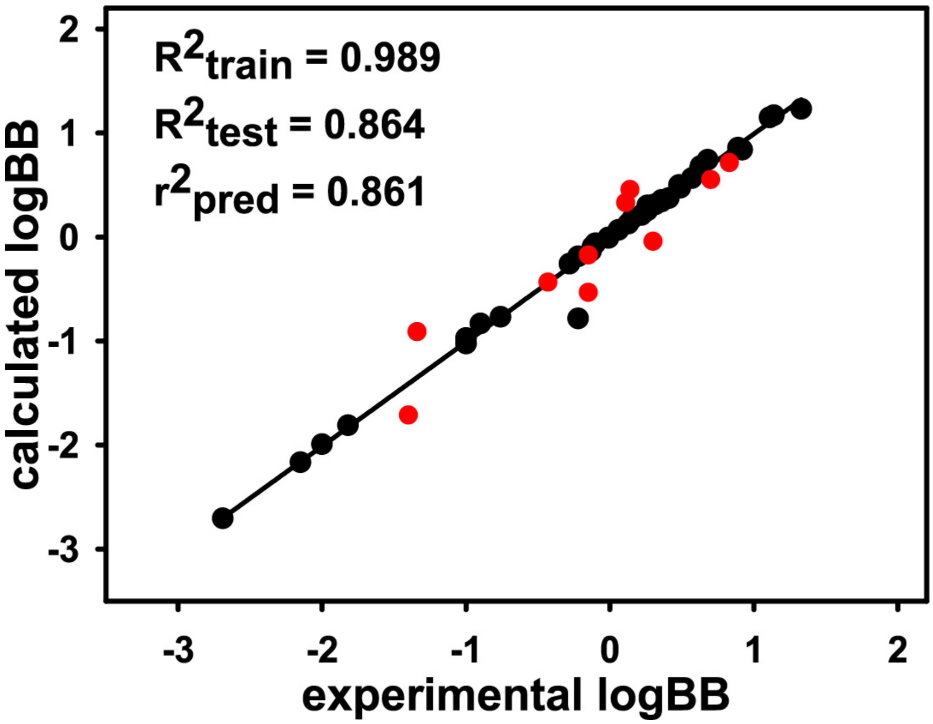

Compounds in the training set: 2477, 2678, 3152, 3559, 3658, 4046, 4078, 4893, 5265, 5339, 5452, 5533, 5754, 60464, 72108, 149096, 198752, 204104, 2733526, 3025986, 3052762, 5362391, 6604009, 9821217, 9821511, 9864647, 9907401, 9907628, 9907851, 10091748, 11003252, 11502034, 11531010, 11545677, 14022483, 14022499, 22620050, 22620062, 22620085, 45272895.

Compounds in the test set: 3357, 5002, 5743, 91769, 3946663, 9864646, 10384745, 11653882, 19696721, 22620075.

Figure S17. Linear diagram showing correlation between the experimental and calculated LogBB values of the Subset 6 molecules in the training (black) and the test set (red).

Fold #2

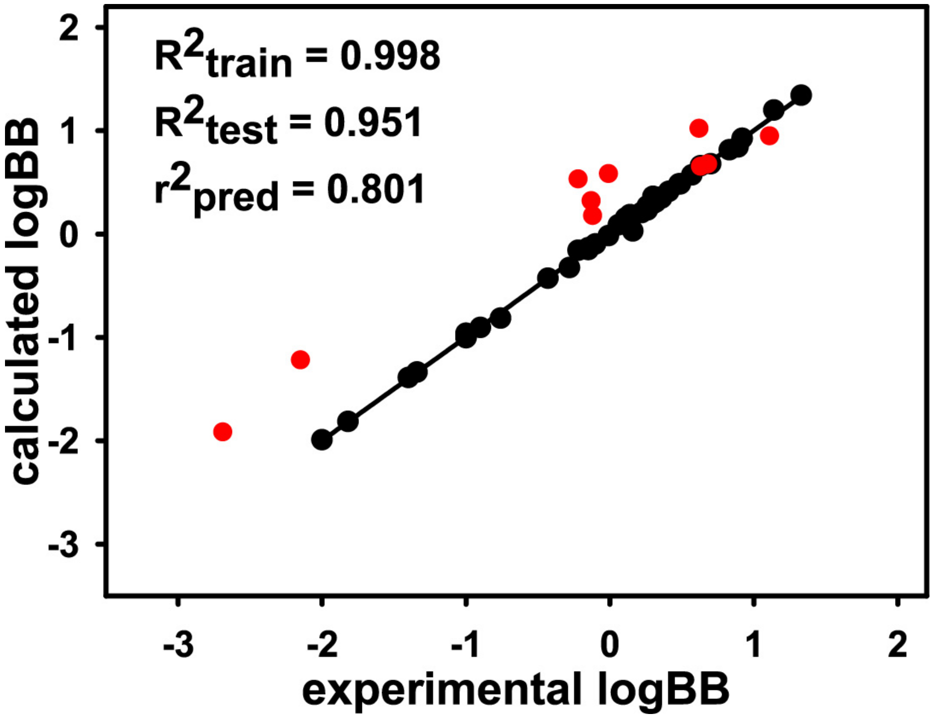

Compounds in the training set: 2477, 3152, 3357, 3559, 3658, 4078, 4893, 5002, 5265, 5452, 5533, 5743, 60464, 72108, 91769, 149096, 198752, 2733526, 3025986, 3052762, 3946663, 5362391, 6604009, 9821511, 9864646, 9864647, 9907401, 9907851, 10091748, 10384745, 11003252, 11531010, 11545677, 11653882, 14022499, 19696721, 22620050, 22620062, 22620075, 45272895.

Compounds in the test set: 2678, 4046, 5339, 5754, 204104, 9821217, 9907628, 11502034, 14022483, 22620085.

Figure S18. Linear diagram showing correlation between the experimental and calculated LogBB values of the Subset 6 molecules in the training (black) and the test set (red).

Fold #3

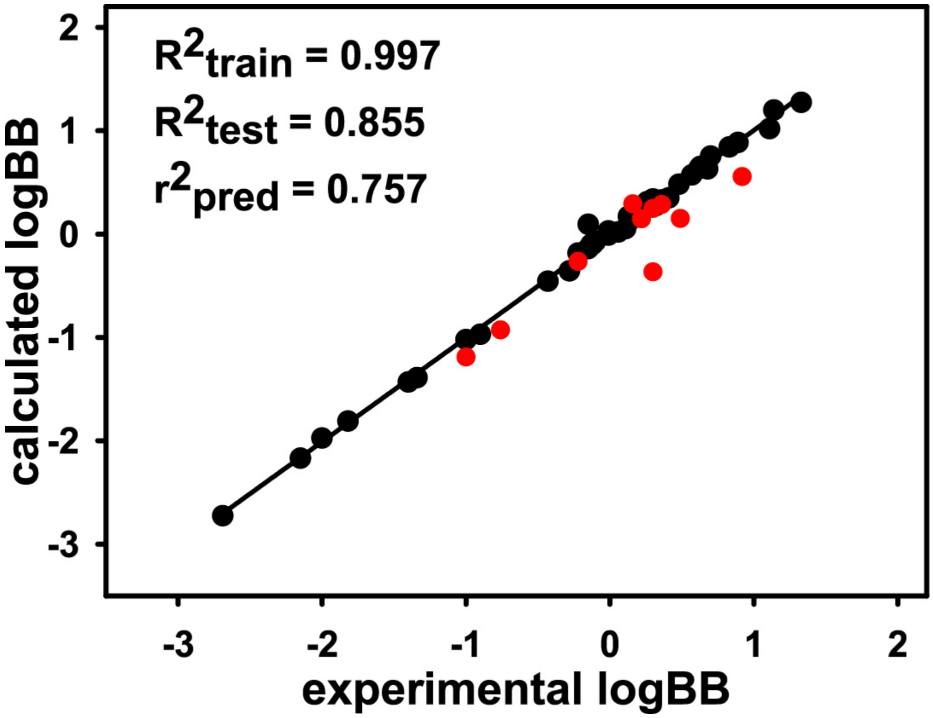

Compounds in the training set: 2678, 3152, 3357, 3559, 4046, 4078, 4893, 5002, 5265, 5339, 5452, 5743, 5754, 60464, 72108, 91769, 198752, 204104, 3025986, 3052762, 3946663, 5362391, 9821217, 9821511, 9864646, 9864647, 9907628, 9907851, 10091748, 10384745, 11502034, 11531010, 11545677, 11653882, 14022483, 19696721, 22620050, 22620075, 22620085, 45272895.

Compounds in the test set: 2477, 3658, 5533, 149096, 2733526, 6604009, 9907401, 11003252, 14022499, 22620062.

Figure S19. Linear diagram showing correlation between the experimental and calculated LogBB values of the Subset 7 molecules in the training (black) and the test set (red).

Fold #1

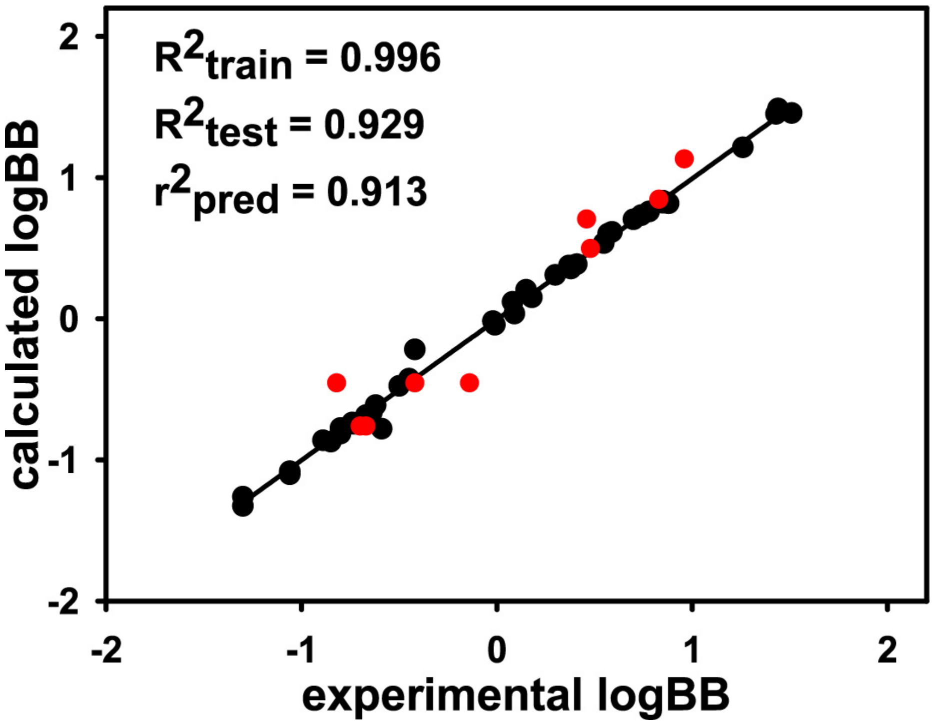

Compounds in the training set: 3151, 3372, 4497, 4748, 5073, 5566, 6167, 31765, 39186, 51263, 51671, 53232, 57347, 59823, 60700, 65998, 71301, 104972, 115237, 123631, 441667, 444031, 456389, 4375468, 5316673, 6603915, 9846311, 9866153, 9931510, 10201984, 10388384, 10884606, 13497176, 14022484, 14022522, 21440942, 22620066, 22620091, 44416780, 70691333.

Compounds in the test set: 12598, 54385, 64761, 119146, 475100, 9909299, 11531660, 19696654, 44592215.

Figure S20. Linear diagram showing correlation between the experimental and calculated LogBB values of the Subset 7 molecules in the training (black) and the test set (red).

Fold #2

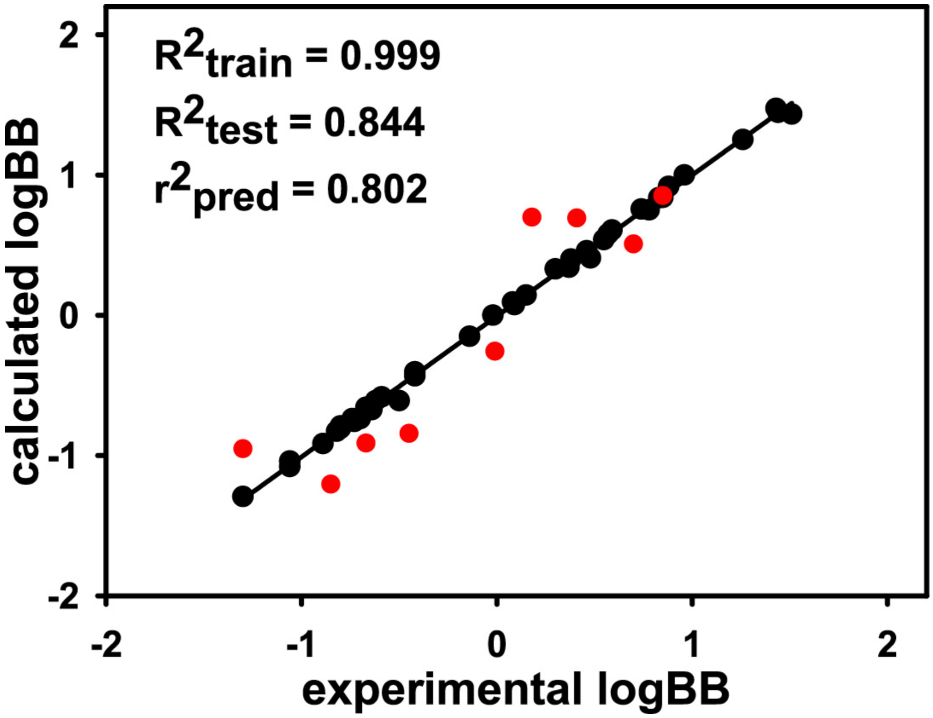

Compounds in the training set: 3372, 4497, 5073, 5566, 6167, 12598, 39186, 51263, 51671, 53232, 54385, 59823, 60700, 64761, 65998, 71301, 104972, 119146, 123631, 441667, 444031, 475100, 4375468, 5316673, 6603915, 9846311, 9909299, 9931510, 10201984, 10388384, 10884606, 11531660, 14022484, 14022522, 19696654, 21440942, 22620066, 44416780, 44592215, 70691333.

Compounds in the test set: 3151, 4748, 31765, 57347, 115237, 456389, 9866153, 13497176, 22620091.

Figure S21. Linear diagram showing correlation between the experimental and calculated LogBB values of the Subset 7 molecules in the training (black) and the test set (red).

Fold #3

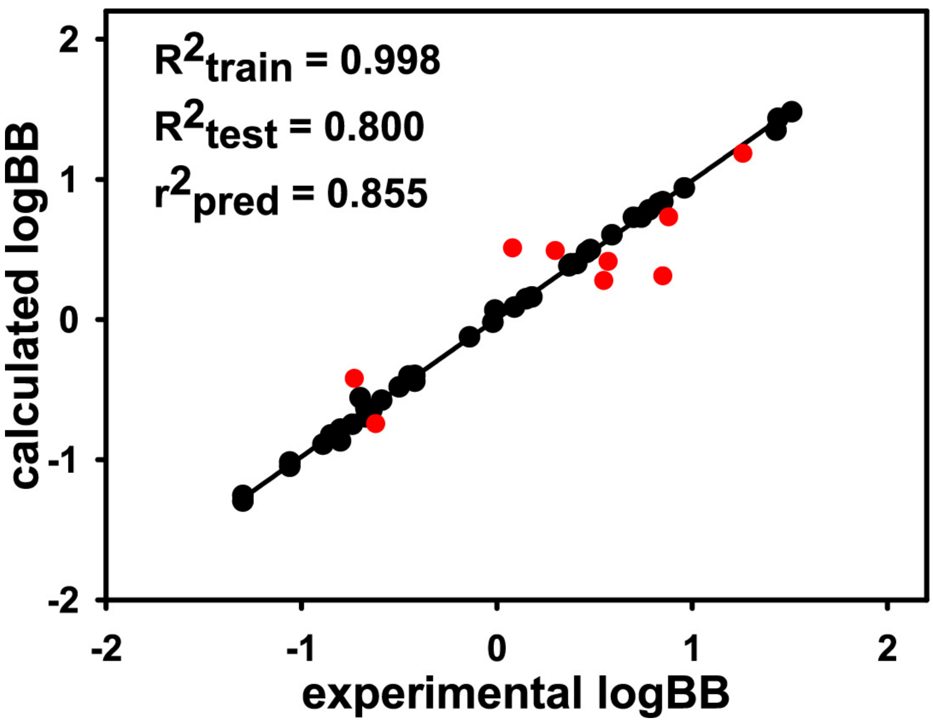

Compounds in the training set: 3151, 3372, 4748, 5073, 5566, 6167, 12598, 31765, 51263, 51671, 54385, 57347, 59823, 60700, 64761, 65998, 104972, 115237, 119146, 123631, 441667, 456389, 475100, 4375468, 6603915, 9846311, 9866153, 9909299, 9931510, 10388384, 10884606, 11531660, 13497176, 14022522, 19696654, 21440942, 22620091, 44416780, 44592215, 70691333.

Compounds in the test set: 4497, 39186, 53232, 71301, 444031, 5316673, 10201984, 14022484, 22620066.

Figure S22. Linear diagram showing correlation between the experimental and calculated LogBB values of the Subset 8 molecules in the training (black) and the test set (red).

Fold #1

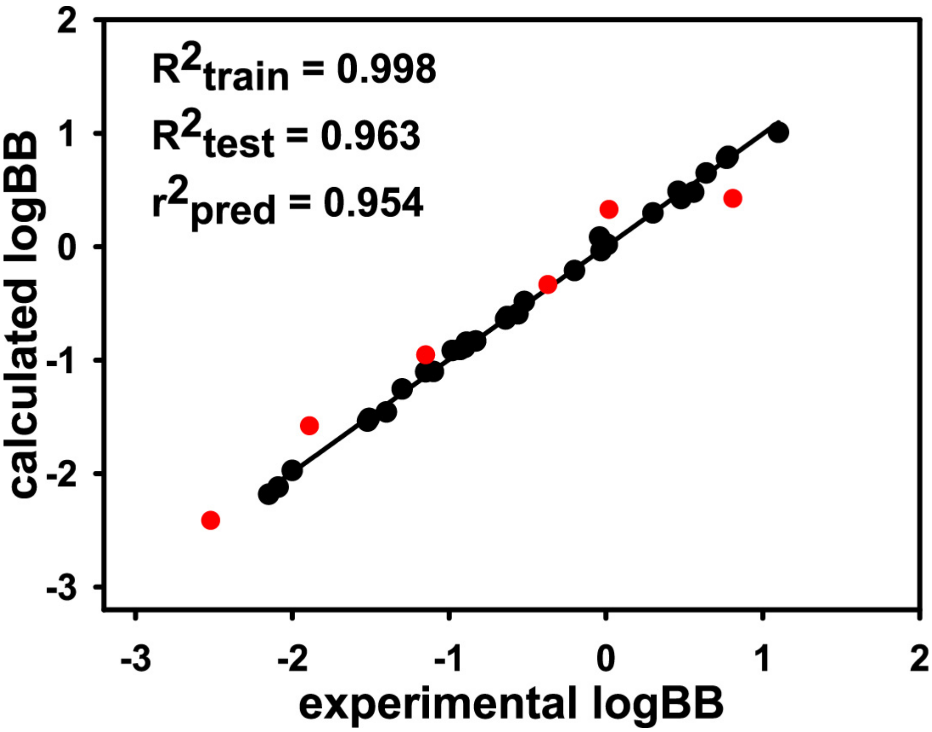

Compounds in the training set: 2247, 2520, 3108, 3230, 3310, 3348, 3488, 3823, 3955, 4112, 5405, 5625, 8223, 31703, 42890, 64143, 65016, 208908, 446156, 5484731, 6426129, 6426143, 6917994, 9806182, 9826520, 9847115, 9916104, 10028436, 11775399, 17905241, 54675783, 57390958.

Compounds in the test set: 47499, 53025, 343473, 19696689, 24894028, 73226213.

Figure S23. Linear diagram showing correlation between the experimental and calculated LogBB values of the Subset 8 molecules in the training (black) and the test set (red).

Fold #2

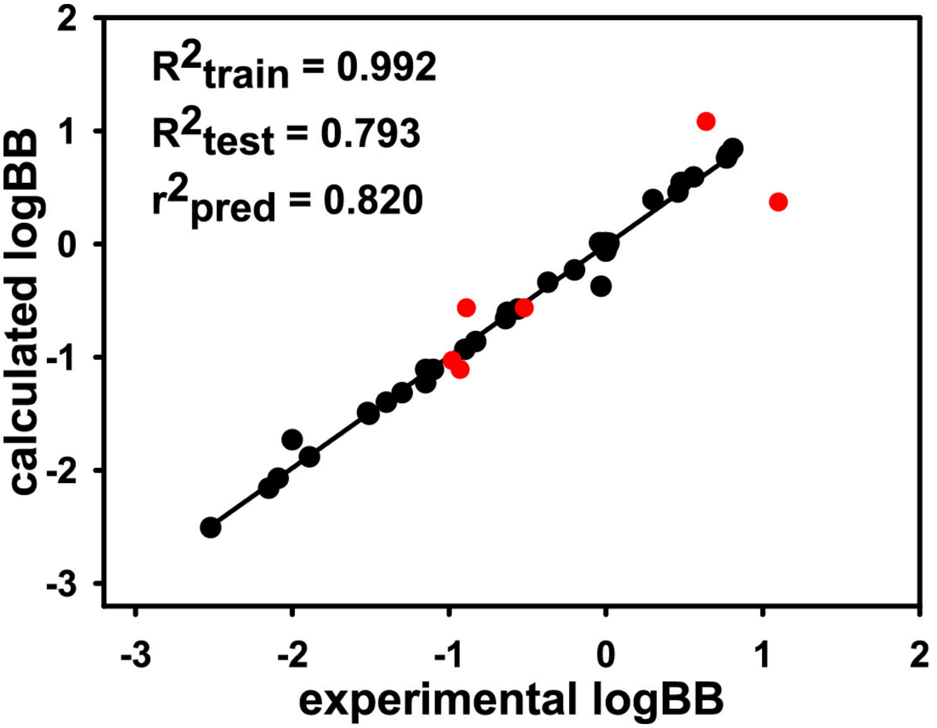

Compounds in the training set: 2520, 3108, 3230, 3310, 3488, 3823, 3955, 4112, 5625, 8223, 31703, 42890, 47499, 53025, 65016, 208908, 343473, 446156, 5484731, 6426143, 6917994, 9806182, 9826520, 9847115, 9916104, 10028436, 11775399, 17905241, 19696689, 24894028, 57390958, 73226213.

Compounds in the test set: 2247, 3348, 5405, 64143, 6426129, 54675783.

Figure S24. Linear diagram showing correlation between the experimental and calculated LogBB values of the Subset 8 molecules in the training (black) and the test set (red).

Fold #3

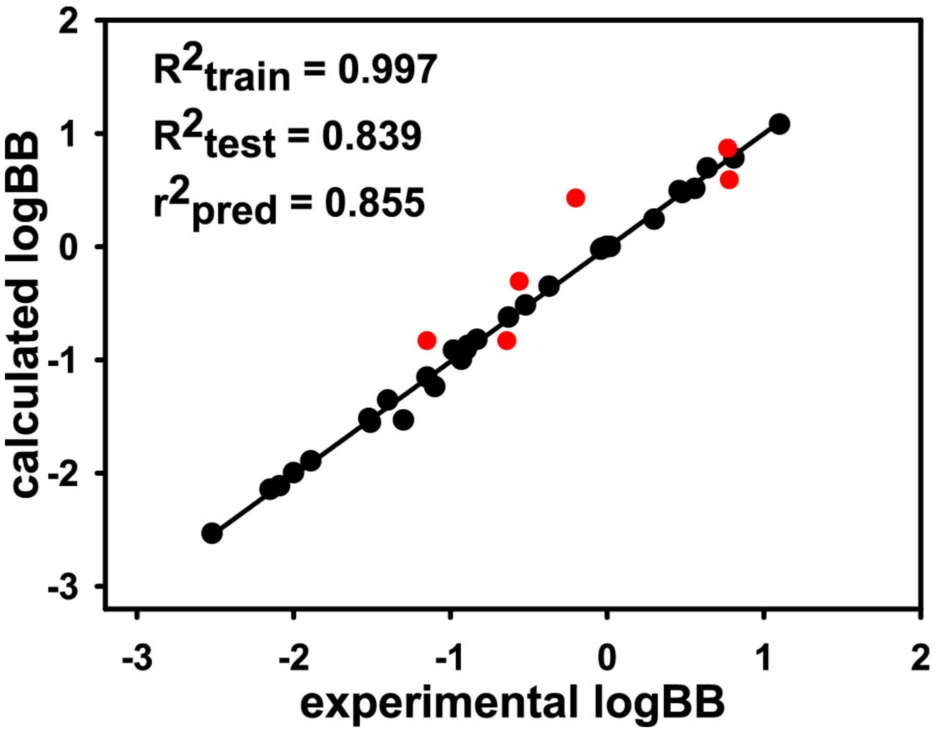

Compounds in the training set: 2247, 3108, 3230, 3310, 3348, 3488, 3823, 4112, 5405, 5625, 8223, 31703, 42890, 47499, 53025, 64143, 208908, 343473, 446156, 5484731, 6426129, 6426143, 6917994, 9806182, 9847115, 9916104, 10028436, 17905241, 19696689, 24894028, 54675783, 73226213.

Compounds in the test set: 2520, 3955, 65016, 9826520, 11775399, 57390958.

v
